# ER-phagy: mechanisms, regulation, and diseases connected to the lysosomal clearance of the endoplasmic reticulum

**DOI:** 10.1152/physrev.00038.2021

**Published:** 2022-02-21

**Authors:** Fulvio Reggiori, Maurizio Molinari

**Affiliations:** ^1^Department of Biomedical Sciences of Cells and Systems, University Medical Center Groningen, University of Groningen, Groningen, The Netherlands; ^2^Institute for Research in Biomedicine, Faculty of Biomedical Sciences, Università della Svizzera italiana, Bellinzona, Switzerland; ^3^School of Life Sciences, École Polytechnique Fédérale de Lausanne, Lausanne, Switzerland

**Keywords:** autophagy, disease, endoplasmic reticulum, ER-phagy, lysosomal degradation

## Abstract

ER-phagy (reticulophagy) defines the degradation of portions of the endoplasmic reticulum (ER) within lysosomes or vacuoles. It is part of the self-digestion (i.e., autophagic) programs recycling cytoplasmic material and organelles, which rapidly mobilize metabolites in cells confronted with nutrient shortage. Moreover, selective clearance of ER subdomains participates in the control of ER size and activity during ER stress, the reestablishment of ER homeostasis after ER stress resolution, and the removal of ER parts in which aberrant and potentially cytotoxic material has been segregated. ER-phagy relies on the individual and/or concerted activation of the ER-phagy receptors, ER peripheral or integral membrane proteins that share the presence of LC3/Atg8-binding motifs in their cytosolic domains. ER-phagy involves the physical separation of portions of the ER from the bulk ER network and their delivery to the endolysosomal/vacuolar catabolic district. This last step is accomplished by a variety of mechanisms including macro-ER-phagy (in which ER fragments are sequestered by double-membrane autophagosomes that eventually fuse with lysosomes/vacuoles), micro-ER-phagy (in which ER fragments are directly engulfed by endosomes/lysosomes/vacuoles), or direct fusion of ER-derived vesicles with lysosomes/vacuoles. ER-phagy is dysfunctional in specific human diseases, and its regulators are subverted by pathogens, highlighting its crucial role for cell and organism life.

## Graphical abstract

**Figure:**
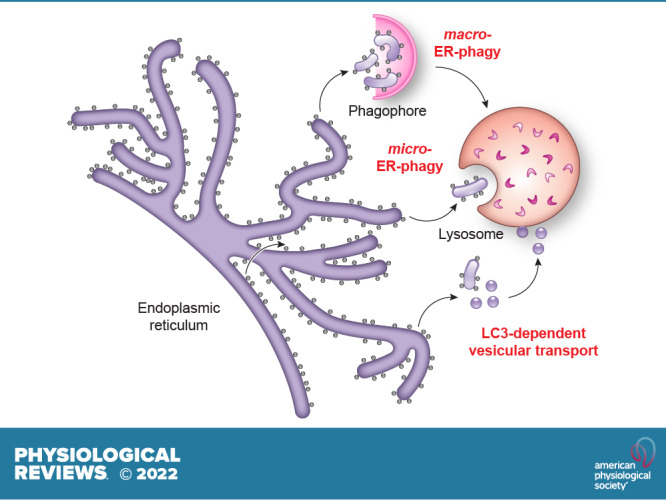

CLINICAL HIGHLIGHTS

ER-phagy is a group of fundamental biological processes that, through distinct mechanisms, lead to lysosomal turnover of selected subdomains of the endoplasmic reticulum (ER). Macro-ER-phagy, micro-ER-phagy, and LC3-dependent vesicular transport play an integral part in the maintenance of ER homeostasis and ultimately guarantee cell adaptations to numerous stresses. The physiological relevance of these pathways is also underscored by human pathologies, including a few neurological disorders and cancers that are caused by mutations in genes involved in ER-phagy. Importantly, the ability of ER-phagy pathways to specifically eliminate misfolded ER proteins, including several disease-associated gene products, provides a potential therapeutic avenue to develop treatments for ER storage disorders. Here, we provide a comprehensive and detailed review of the molecular pathways that mediate ER-phagy and its physiological functions.

## 1. INTRODUCTION

### 1.1. Endoplasmic Reticulum, Lysosomes, and Autophagy: a Historical Perspective

The endoplasmic reticulum (ER) is an organelle present in all eukaryotic cells (1). ER size and activity are increased upon induction of unfolded protein responses (2–4) and are reduced upon activation of ER-phagy responses (5). These anabolic and catabolic pathways adapt protein, lipid, and oligosaccharide biogenesis and content to cellular demands, control calcium homeostasis and signaling, ensure clearance of toxic components, and respond to pathogen invasion or disease states. A search in the PubMed database reveals that >100,000 publications, 10% of which are reviews, cover each one of the established functions of the ER. The same holds true when the keyword used to inspect the PubMed database is “lysosome,” defined as a membrane-bound organelle containing hydrolytic enzymes and characterized by an acidic luminal pH (6), or “autophagy,” which refers to the lysosomal consumption of unwanted or dysfunctional cell-own components (7, 8). Thus, the reader will certainly find recent reviews that proficiently cover each aspect of ER, lysosomes, or autophagy in health and disease, concepts that have already entered the textbooks (9). In this introduction, for the sake of conciseness, we report on the pioneering work of scientists, most of them working at the Rockefeller University in the midst of the last century, who discovered and gave a relevant impulse to the initial morphological and functional characterization of both the ER and the endolysosomal compartments and reported on autophagic events that cells activate to eventually degrade their own content, including organelles such as the ER.

### 1.2. The Discovery of the Endoplasmic Reticulum

The ER was first observed in light microscopy by Bernhard Solger, who called it “basal filaments” (10), and subsequently by Charles Garnier, who named it “ergastoplasm” and predicted its involvement in secretion (11). Further structural and functional characterization of the ergastoplasm had to wait for five decades and the introduction of both ultracentrifugation and electron microscopy (EM). By sequential centrifugations, the Belgian scientist Albert Claude separated mitochondria from smaller particles that he named “microsomes” (12). The intensive red staining obtained by supplementing these fractions with pyronine showed that most of the cytosolic ribonucleic acid was associated with microsomes and offered a first link between microsomes and protein synthesis (13). The abundant incorporation of labeled amino acids in proteins isolated from the microsomal fraction corroborated this hypothesis (14, 15). Analysis of purified microsomes by electron microscopy initially provided little or no information. Thus, Keith Porter proposed the use of cultured cells as specimens for electron microscopy and produced the first micrographs showing a lacelike structure throughout the cytoplasm (16). Keith Porter improved the quality of the images by preparing thin, serial cell sections, speculated that the lacelike structures could be a center for protein synthesis, and eventually changed the name of the organelle from ergastoplasm to “endoplasmic reticulum” (17, 18). Electron microscopy examination of cells and isolated microsomal fractions revealed small electron-dense structures adjacent to the ER membrane (19), which were eventually termed “ribosomes” at the first symposium of the Biophysical Society held at the Massachusetts Institute of Technology in 1958. Electron microscopy also revealed that the ER consists of both large and flattened cisternae and tubular elements (20–22).

### 1.3. The Discovery of Lysosomes and of Lysosomal Clearance of Cell-Own Components

By the end of the nineteenth century, it had already been proposed that intracellular digestion proceeds at acidic pH (23). The discovery of lysosomes, however, had to wait for half a century. In contrast to the ER and other organelles such as mitochondria and the Golgi apparatus, which were identified by standard cytological approaches, the lysosome was identified through its function and by serendipity. It was the unexplained low activity of acid phosphatase detected upon gentle cell fractionation and its much higher activity recovered when cells were harshly broken with a Waring blender that led Christian de Duve to hypothesize that this hydrolytic enzyme is protected within an organelle, possibly the mitochondrium (24). The rupture of the laboratory high-speed centrifuge and the consequent change in the cell fractionation protocol, here the serendipity, revealed that the acid phosphatase and other hydrolytic enzymes are in organelles lighter than mitochondria (25). These organelles were named “lysosomes” (26, 27). Light and electron microscopy contributed to the further characterization of these degradative compartments (28) and to their differentiation according to the exogenous (heterophagy) or endogenous (autophagy) material that they are digesting (7, 29). Nowadays, lysosomes are defined as storage organelles for hydrolytic enzymes, which eventually fuse with endosomes or autophagosomes, in the latter case to generate degradative compartments named autolysosomes (6, 30). They are considered a final station, in which cellular components to be turned over are eventually delivered, as well as crucial signaling hubs that modulate protein, lipids, ions, and stress and nutrient dynamics (31, 32). Crucial for the context of this review are the numerous observations demonstrating bulk but also selective transport and digestion of cytoplasmic content, including organelles, within lysosomes, autolysosomes, or vacuoles. These pathways were initially named “focal autolysis” (33) or “focal cytoplasmic degradation” (34), but they eventually made history as autophagy processes (8).

### 1.4. Capturing Organellophagy on Micrographs: Original Reports and Predictions

Organelle clearance within acidic degradative compartments was morphologically characterized by cell biology pioneers starting in the mid-1950s. ER, mitochondria, and other subcellular organelles were mostly seen within single (rarely double)-membrane organelles that were called “cytolysomes” (35) or autophagic vacuoles (8). Analysis of the micrographs immediately raised questions such as “how do organelles get access to the lumen of degradative compartments?” and “are organelles selectively or nonselectively taken up by degradative compartments?” (7, 8, 34, 36).

In response to the first question, micrographs caught endosomes and/or lysosomes in the act of capturing organelles, upon inward budding of their limiting membranes (7, 37). Contemporary knowledge would classify these processes as micro-autophagy (38, 39), which is further defined by the engulfed organelle, e.g., micro-mitophagy and micro-ER-phagy. The interpretation of other images, in which organelles were detected within the degradative compartments, was less clear and left some mechanistic questions, especially regarding whether the organelles *1*) were incorporated via phagocytosis; *2*) entered from the cytoplasm through holes in the lysosomal membrane that were then rapidly resealed; or *3*) were captured by a “saclike structure” forming around them, which was subsequently filled with hydrolytic enzymes. The first option was rapidly dismissed because organelles were also seen in autophagic vacuoles (hereafter “autolysosomes” as per current nomenclature) of cells that lack phagocytic activity. The second scenario seemed unlikely. The third, which was proposed by Alex Novikoff (40), would nowadays be classified as macro-autophagy, and termed, for example, macro-mitophagy and macro-ER-phagy when the “saclike structures” sequester mitochondria or ER, respectively.

In response to the second question about selectivity of organelle capture by degradative compartments, Christian de Duve and Robert Wattiaux, in their seminal review on the function of lysosomes, hypothesized on the stochastic nature of the process (7). The main reason behind this notion was that individual autolysosomes often contain both ER and mitochondria fragments as well as other cytoplasmic components (7). Indications that the organellophagy process could actually be selective emerged, for the ER, from images revealing the exclusive presence of ER fragments inside autolysosomes in the fat body of insects during the formation of storage granules (41), in rat hepatocytes after cessation of treatment with phenobarbital, an antiepileptic drug that triggers ER stress (42), and in pancreatic cells from guinea pig injected with cobalt chloride to induce massive intra-ER deposition of digestive enzymes (43).

Eventually, more recent investigations have revealed that stimuli such as those triggered by either nutrient deprivation or chemically induced ER stresses elicit an autophagic degradation of cell-own cytoplasmic content, including the ER, without an apparent specificity (pleiotropic stimuli; sect. 3.3.1). Then, there are cues such as recovery from ER stress, the luminal accumulation of misfolded proteins, stalling of ribosomes, and pathogen invasion, which induce the selective turnover of subdomains of the ER by autophagy (ER-centric stimuli; sect. 3.3.2) (5, 44–47). In this review, we provide a detailed overview of how research performed in the last few years impacts on the fundamental morphological and functional observations made by cell biology pioneers like Alex Novikoff, Christian de Duve, and many others, who reported on organelle delivery within lysosomal compartments for clearance more than 50 years ago. Specifically, after an introduction to the mechanistic principles that govern autophagy pathways and the selectivity within these processes (sect. 2), we cover the current knowledge on lysosomal- or vacuolar-controlled ER degradation, which has been named reticulophagy or ER-phagy (the latter name is used throughout our text) (sect. 3). ER-phagy is emerging as a crucial catabolic pathway that *1*) contributes with other autophagic pathways to the rapid mobilization of nutrients during cell starvation, *2*) constitutively and actively regulates size and shape of the ER, *3*) ensures clearance of ER subdomains containing aberrant or aged proteins and lipids, *4*) is coopted by pathogens to invade host cells, and *5*) misfunctions in an increasing list of diseases. The work of the new generation of cell biologists and biochemists is adding more and more precise molecular details of the different pathways that directly link the ER, the major biosynthetic compartment of the eukaryotic cell, with the lysosome, its principal catabolic compartment. The relevance of this intimate connection is not just exquisitely cell biological, as recent evidence highlighted in sect. 4 is revealing its important medical implications (47, 48).

## 2. AUTOPHAGY AND SELECTIVE AUTOPHAGY

### 2.1. Types of Autophagy

Over the past 25 years, a multitude of seminal discoveries have made autophagy a prominent research area in life sciences and medicine. The processes encompassed by this term participate in key cellular and organismal functions such as response to shortage of nutrients, development and cell differentiation, degradation of aberrant or obsolete structures, life span extension, immunity, and programmed cell death (49–53). Autophagy also plays a relevant role in the pathophysiology of neurodegenerative, cardiovascular, chronic inflammatory, muscular, and autoimmune diseases and some malignancies (54, 55). Consistently, modulation of autophagy is a promising strategy for development of effective therapies to prevent or cure specific pathological conditions (52, 56, 57).

**FIGURE 1.**
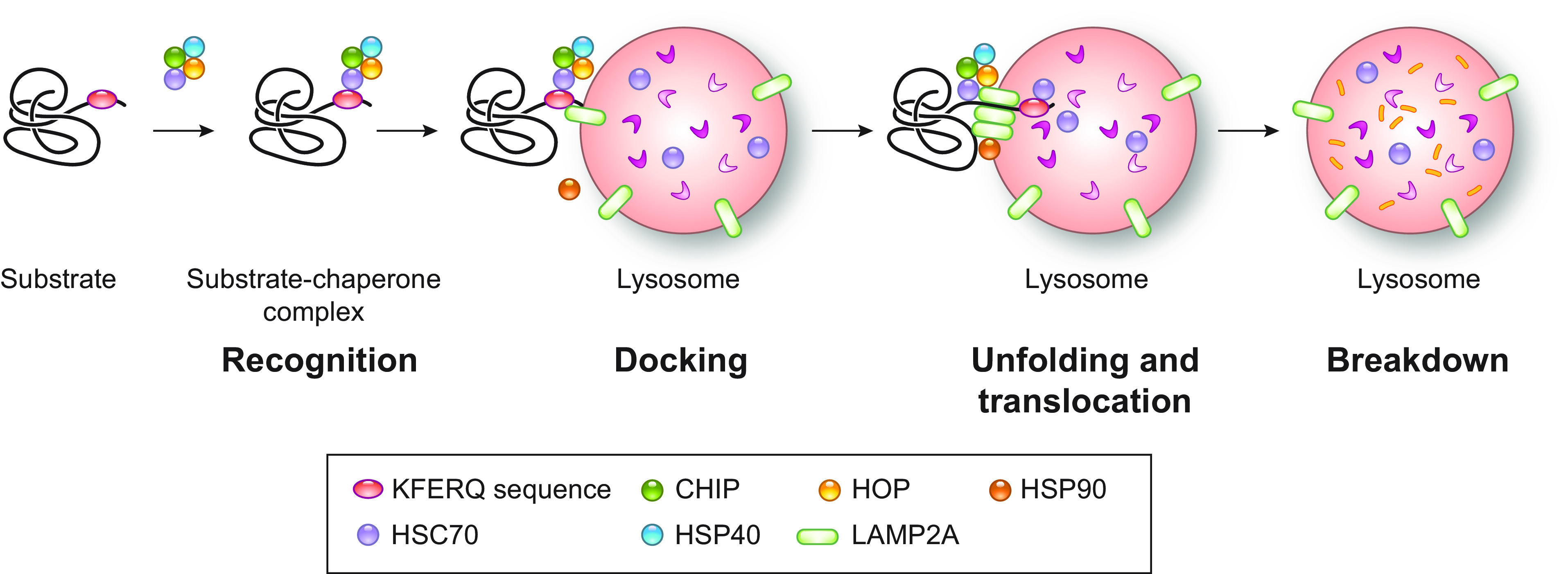
The mechanism of CMA. During CMA, KFERQ or KFERQ-like motifs are recognized by the cytosolic chaperone heat shock cognate 71-kDa protein (HSC70) and its cochaperones, including HSC70-interacting protein (CHIP), heat shock protein (HSP40), and HSC70–HSP90 organizing protein (HOP), leading to the assembly of a cargo-chaperone complex. This complex engages LAMP2A at lysosomes, and substrates are translocated into the lysosomal lumen via a process involving the multimerization of LAMP2A and lysosomal HSC70. Substrates are then broken down by lysosomal proteases, while the LAMP2A multimers disassemble for reuse. See glossary for abbreviations.

**FIGURE 2.**
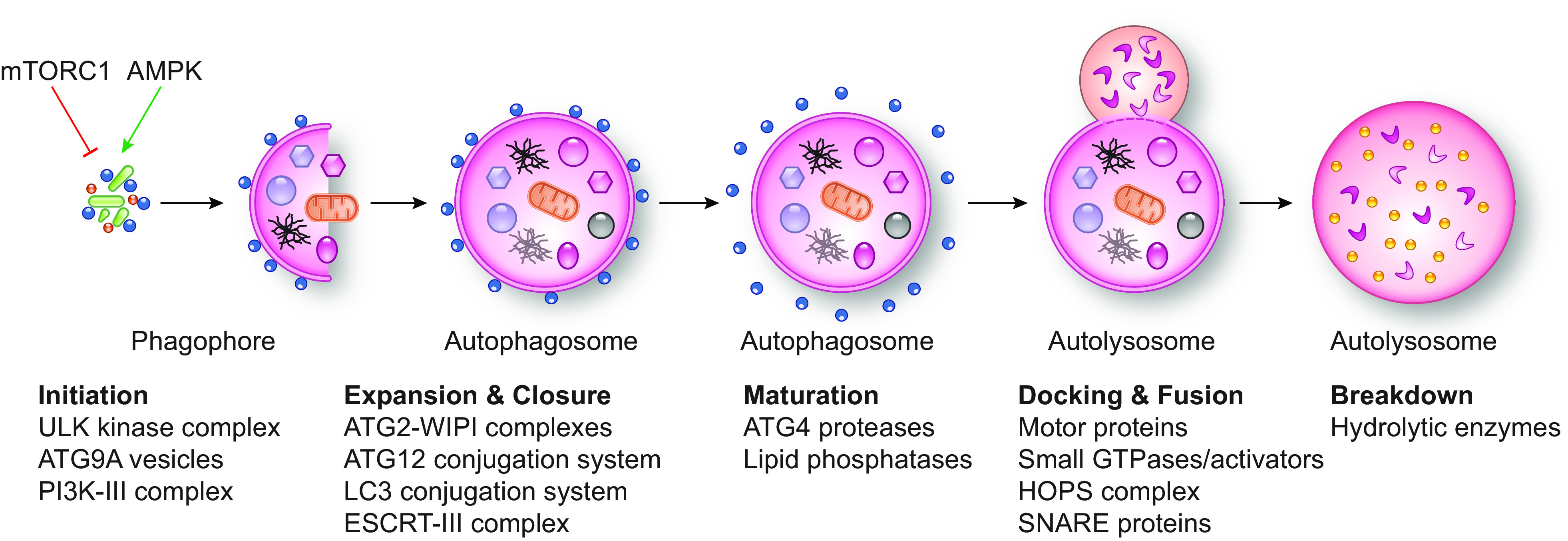
The mechanism of macro-autophagy. The induction of macro-autophagy, often modulated by mTORC1 and/or AMPK, leads to the activation of the ULK kinase complex and the subsequent formation of the phagophore. The nucleation of the phagophore requires both the fusion of Atg9/ATG9A-positive vesicles with possibly other ones and the activity of the macro-autophagy-specific PI3K-III complex. The subsequent recruitment and engagement of the ATG2 proteins and the 2 Ub-like conjugation systems (but also other factors) drives the phagophore expansion and its closure into an autophagosome. Autophagosome maturation is characterized by the release in the cytoplasm of the components intervening during autophagosome biogenesis and the recruitment of factors mediating the docking and fusion with late endosomes and/or lysosomes. Exposure of the autophagosomal cargo to the lysosomal hydrolyses leads to its breakdown into basic metabolites. The major players mediating autophagosome biogenesis and consumption are indicated below each step of autophagy. See glossary for abbreviations.

**FIGURE 3.**
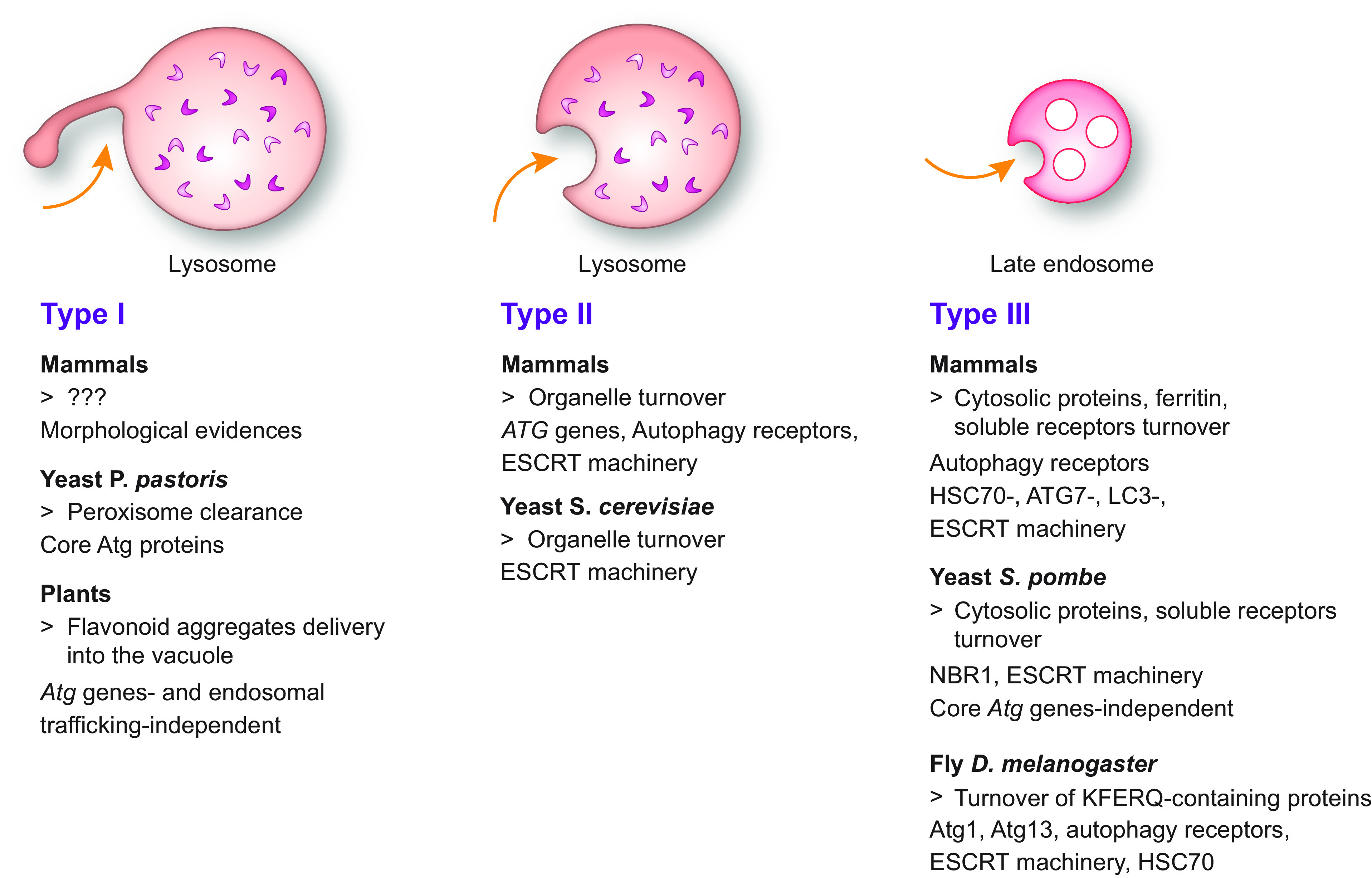
The mechanisms of micro-autophagy. During micro-autophagy, the cargo is directly engulfed by late endosomes and/or lysosomes/vacuoles. Micro-autophagy pathways are subdivided into type I, in which the lysosome/vacuole extrudes membrane projections that enwrap the cargo; type II, in which the lysosome/vacuole engulfs the cargo; and type III, in which the cargo is sequestered by late endosomes. Orange arrows indicate where the targeted cytoplasmic cargoes are engulfed. See glossary for abbreviations.

Currently, three main types of autophagy are recognized in mammals: macro-autophagy, micro-autophagy, and chaperone-mediated autophagy (CMA) (FIGURES 1–3). CMA is a selective type of autophagy principally dedicated to chaperone-assisted clearance of cytosolic proteins possessing a KFERQ or a KFERQ-like motif in their amino acidic sequence (58). The substrates are translocated one by one into the lysosomal lumen, via a channel formed by multimerization of the lysosomal integral membrane protein lysosomal-associated membrane protein 2A (LAMP2A) (FIGURE 1) (59–63).

CMA only operates in birds and mammals (60). In contrast, macro- and micro-autophagy (FIGURES 2 and 3, respectively) operate in all Eukarya to remove macromolecules, portions of organelles, and pathogens (64, 65). Macro- and micro-autophagy are defined as bulk or nonselective when the cargo destined for turnover is heterogeneous in composition and appears to be captured randomly. They are considered selective when a distinct cargo is exclusively or preferentially targeted (66).

#### 2.1.1. Bulk autophagy.

##### 2.1.1.1. bulk macro-autophagy.

Genetic screens in the yeast *Saccharomyces cerevisiae*, starting from the beginning of the 1990s, have led to the identification of >40 *ATG* genes, whose products are involved in a variety of selective and nonselective autophagic pathways (65). Around 20 ATG proteins compose the highly conserved core machinery that controls autophagosome biogenesis in all eukaryotes.

The proteins encoded by these genes have been divided into six functional modules: the Atg1/ULK kinase complex, the macro-autophagy-specific class III phosphatidylinositol 3-kinase (PI3K-III) complex, the Atg9/ATG9A-positive vesicles, the Atg2/ATG2-Atg18/WIPI complex, and the Atg12/ATG12 and the Atg8/LC3 conjugation systems (TABLE 1) (65, 67). The rest of the ATG proteins are organism specific or drive selective types of autophagy. For example, Atg23 and Atg27 are organism specific, as they form a functional complex with Atg9 in yeast (68–70), whereas yeast Atg32 and Atg33 regulate selective removal of mitochondria by macro-mitophagy (71). Of note, there are forms of macro-autophagy that do not require the function of all ATG modules, and these have been defined as unconventional (72, 73). Conversely, single or groups of ATG proteins operate in other cellular processes, including micro-autophagy (see below), and these are defined as unconventional functions of the ATG proteins (72, 73).

**Table 1.** ATG protein functional modules and their components

	Yeast *S. cerevisiae*	Mammalian
Atg1/ULK kinase complex	Atg1	ULK1 or ULK2
	Atg11	FIP200/RB1CC1
	Atg13	ATG13
	Atg17	
	Atg29	
	Atg31	
PI3K-III complex		ATG101
	Atg6	BECN1
	Atg14	ATG14L/Barkor
	Atg38	NRFB2
	Vps15	p150
	Vps34	VPS34
Atg9/ATG9A vesicles	Atg9	ATG9A
	Atg23	
	Atg27	
Atg2/ATG2-Atg18/WIPI complexes	Atg2	ATG2A or ATG2B
	Atg18	WIPI4 (possibly WIPI3)
Atg12/ATG12 and Atg8/LC3 conjugation systems	Atg3	ATG3
	Atg4	ATG4A to ATG4D
	Atg5	ATG5
	Atg7	ATG7
	Atg8	LC3A, LC3B, LC3C, GABARAP, GABARAPL1, and GABARAPL2
	Atg10	ATG10
	Atg12	ATG12
	Atg16	ATG16L1
	Atg21	WIPI2

See glossary for abbreviations.

Autophagosome biogenesis and consumption can be divided into five discrete steps, each of which involves a specific set of core ATG proteins, but also other factors (65, 67) (FIGURE 2):

1. The initiation step is characterized by the activation of the Atg1/ULK kinase complex, which initiates the formation of the phagophore (or isolation membrane), the precursor structure of the autophagosome (FIGURE 2) (65, 67). As a matter of fact, multiple signaling cascades regulating macro-autophagy act on this complex (74, 75). For example, the activity of the Atg1/ULK kinase complex is repressed upon phosphorylation of various ULK complex subunits by the mammalian target of rapamycin complex 1 (mTORC1) complex. The kinase activity of mTORC1 is promoted by a variety of upstream signals including growth factors and nutrients such as amino acids (76). In the absence of the aforementioned signals, the kinase activity of mTORC1 is inhibited, the components of the ULK complex are dephosphorylated, and this initiates macro-autophagy (74, 75). The ULK kinase complex is also positively regulated by 5′ AMP-activated protein kinase (AMPK), which senses the cellular energy status and is activated when intracellular AMP increases, reflecting a decrease in availability of ATP (74, 75). Phosphorylation and dephosphorylation events also modulate the supra-assembly of numerous Atg1/ULK kinase complexes via liquid phase separation (77). The nucleation of the phagophore is initiated by heterotypic fusion of Atg9/ATG9A-positive vesicles with vesicles that are probably derived from multiple membrane sources, including recycling endosomes and ER (78). The macro-autophagy-specific class III phosphatidylinositol 3-kinase (PI3K-III) complex also participates in phagophore nucleation by catalyzing the synthesis of phosphatidylinositol-3-phosphate (PtdIns3P) on autophagosomal membranes, which is important for the recruitment of additional components of the ATG machinery (FIGURE 2).
2. The expansion step relies on the association to the phagophore of ATG machinery components such as the Atg2/ATG2-Atg18/WIPI complex and the Atg12/ATG12 and Atg8/LC3 conjugation systems that eventually conjugate the members of the ubiquitin (Ub)-like Atg8 protein family (in human LC3A, LC3B, LC3B, GABARAP, GABARAPL1, and GABARAPL2) to the phosphatidylethanolamine present in the membrane of the growing phagophore (FIGURE 2). The ATG2-WIPI complexes appear to be key in supplying at least part of the lipids required for the phagophore expansion through their direct transfer from the ER (79–82) in conjunction with Atg9/ATG9A lipid scramblase activity (83–85). In contrast, the two conjugation systems marginally participate in this process. Rather, they are crucial for the closure of the phagophore into an autophagosome (86, 87). This latter step also requires the ESCRT-III complex (88–91). Autophagosomes typically have a diameter of 500–800 nm that can increase to accommodate larger cargoes.
3. The maturation step is characterized by the dissociation and release in the cytoplasm for reutilization of the components intervening during autophagosome biogenesis (92). Two important aspects of this step are the hydrolysis of PtdIns3P into phosphatidylinositol by phosphatases from the myotubularin family (93, 94) and the deconjugation of the Atg8/LC3 proteins from their lipid anchor by Atg4/ATG4 cysteine proteases (FIGURE 2) (95–97).
4. The docking and fusion step involves motor proteins that ensure the encounter between autophagosomes generated throughout the cytoplasm and lysosomes located in the perinuclear region (98) (FIGURE 2). Kinesin motors and the small GTPase ARL8 regulate the anterograde transport that allows lysosomes to dock to peripheral autophagosomes. Retrograde transport is regulated by dynein-dynactin motors, the small GTPase Ypt7/RAB7, and the LC3-binding nucleotide exchanger factors Ccz1/CCZ1-Mon1/MON1A (99, 100). The coupling of anterograde and retrograde transport and autophagosome-lysosome fusion through a kiss-and-run mechanism involves the homotypic fusion and protein sorting (HOPS) complex. This is recruited at the membrane of endosomes and lysosomes upon association with RAB7 and PtdIns3P or at the membrane of autophagosomes upon association with soluble *N*-ethylmaleimide-sensitive factor attachment protein receptors (SNAREs) and other tethering factors (92, 101, 102).
5. The breakdown step consists of the lysis of the inner autophagosomal membrane and the turnover of the cargo by lysosomal hydrolytic enzymes including proteases, lipases, glycosidases, nucleases, and phosphatases (FIGURE 2). This event generates the basic metabolites that are subsequently transported into the cytoplasm or connected organelles, for their use as either building blocks for the synthesis of new macromolecules or energy source (53, 103).

##### 2.1.1.2. bulk micro-autophagy.

In micro-autophagy, the cargo is directly engulfed by late endosomes and/or lysosomes/vacuoles (FIGURE 3) (64). Micro-autophagy pathways are categorized into three types: type I, in which the lysosome/vacuole extrudes membrane projections that enwrap the cargo; type II, in which the lysosome/vacuole engulfs the cargo; and type III, in which the cargo is engulfed by late endosomes (104) (FIGURE 3).

In mammals, type I micro-autophagy has only been characterized at the morphological level (105–109). In the yeast *Pichia pastoris* it is involved in peroxisome clearance (104, 110–115), and in plants it delivers anthocyanins and other flavonoid aggregates into the vacuole (116) (FIGURE 3). Both in mammalian and yeast cells, type II micro-autophagy regulates organellar turnover (see, e.g., Refs. 113, 117–120) (FIGURE 3). Type III micro-autophagy is operated by late endosomes and ensures turnover of cytosolic proteins, ferritin, and soluble autophagy receptors in mammalian cells (121–124), of cytosolic proteins and soluble autophagy receptor in the yeast *Schizosaccharomyces pombe* (125), and of KFERQ-tagged proteins in *Drosophila melanogaster* (126).

#### 2.1.2. Selective autophagy.

Macro- and micro-autophagy have been considered for a long time nonselective processes for bulk degradation of cytoplasmic components. During the last decade, however, it has become clear that they also contribute to selective autophagy. Selective autophagy is defined as the lysosomal clearance of defined intracellular components sequestered into autophagosomes or directly delivered to endolysosomal degradative compartments (66, 127). Selective autophagy maintains organismal physiology upon regulated and selective turnover of portions of organelles including mitochondria (mitophagy), lipid droplets (lipophagy), lysosomes (lysophagy), peroxisomes (pexophagy), nucleus (nucleophagy), and the ER (ER-phagy) but also large protein/RNA complexes such as ribosomes, midbodies, and inflammasomes, as well as single proteins like the transcription factor GATA4, aggregated IRE1, and others (sect. 3) (5, 66, 127–138).

##### 2.1.2.1. general characteristics of selective autophagy receptors.

**FIGURE 4.**
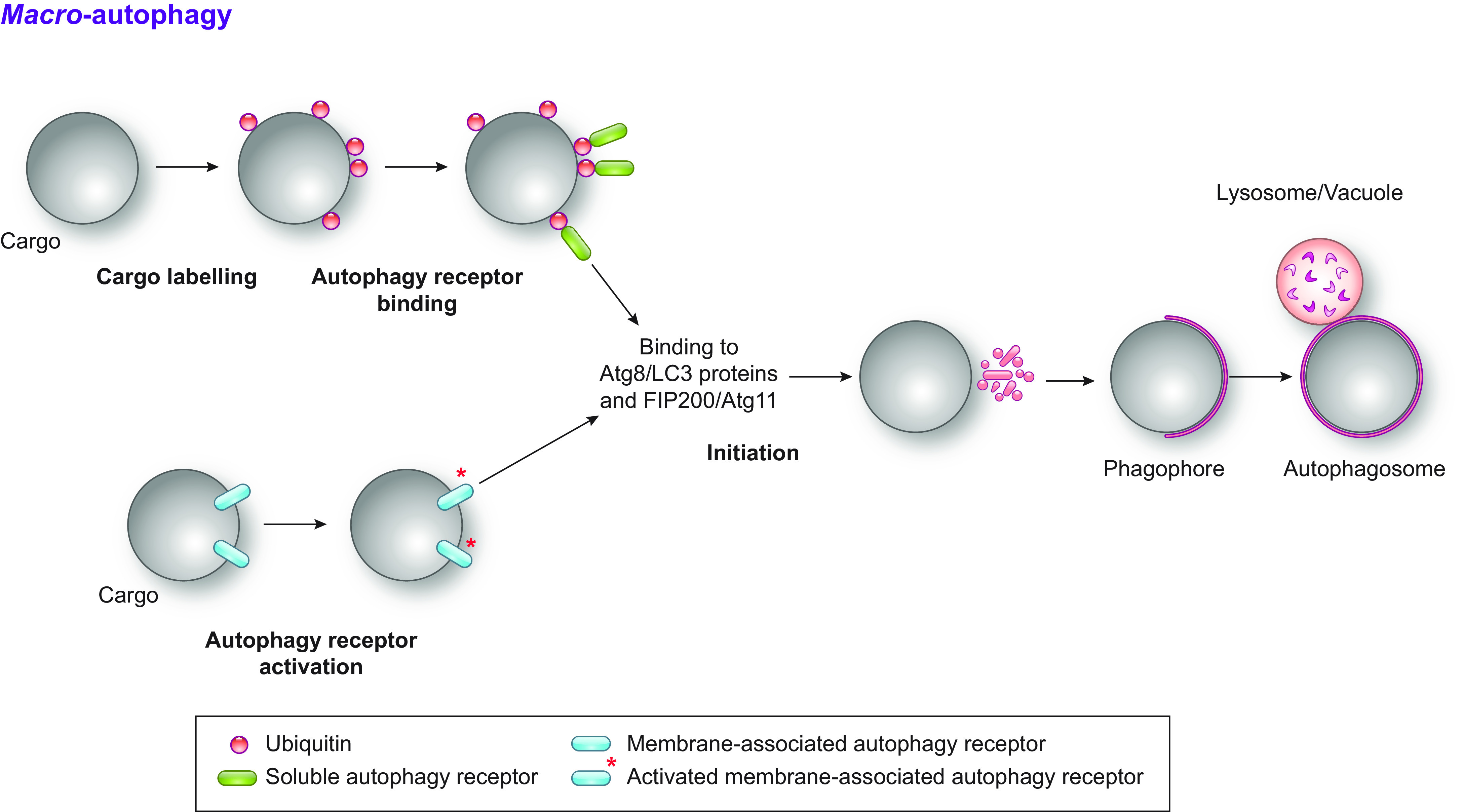
Selective macro-autophagy. Selective types of macro-autophagy are initiated through 2 different mechanisms. In the first, ubiquitylation of the cargo triggers the binding of soluble autophagy receptors possessing an ubiquitin-binding domain, which in turn recruits both Atg8/LC3 proteins and FIP200 (or its yeast counterpart Atg11). This leads to the assembly and activation of the ATG machinery, which mediates the local formation of a phagophore and its expansion around the cargo. In the second mechanism, autophagy receptors embedded into the limiting membrane of a targeted organelle are activated, often through posttranslational modifications such as phosphorylation, which primes the organelle for degradation. Activated autophagy receptors recruit Atg8/LC3 proteins and FIP200/Atg11, and the local formation and expansion of the phagophore leads to the sequestration of the targeted organelle into an autophagosome.

**FIGURE 5.**
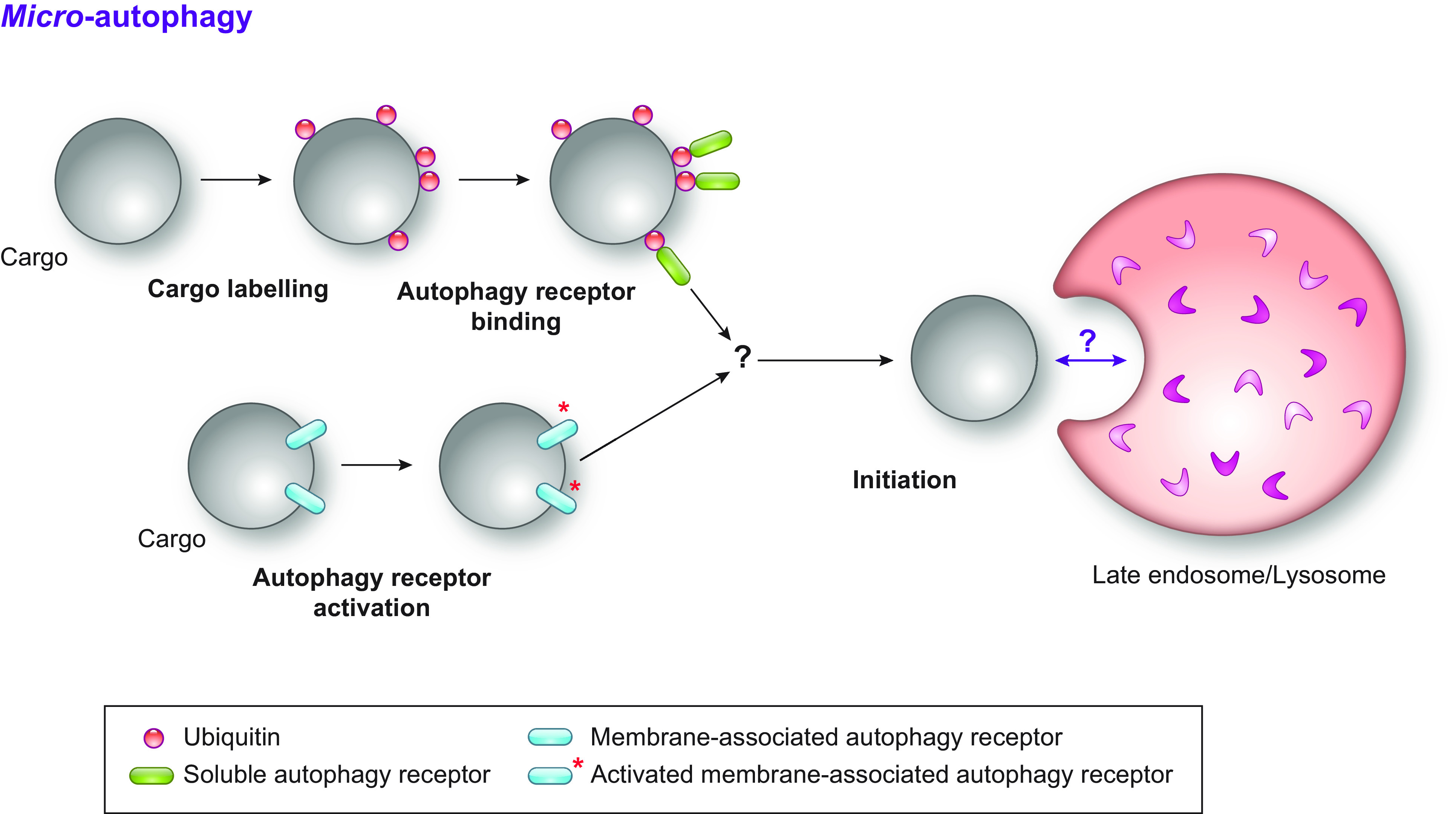
Selective micro-autophagy. Autophagy receptors can also mediate micro-autophagy upon their activation, possibly through direct or indirect binding to the cargo. In this context, it remains unknown how the autophagy receptors are recognized by endolysosomal compartments and trigger the engulfment of the cargo. There might be forms of micro-autophagy that do not require conventional autophagy receptors.

**FIGURE 6.**
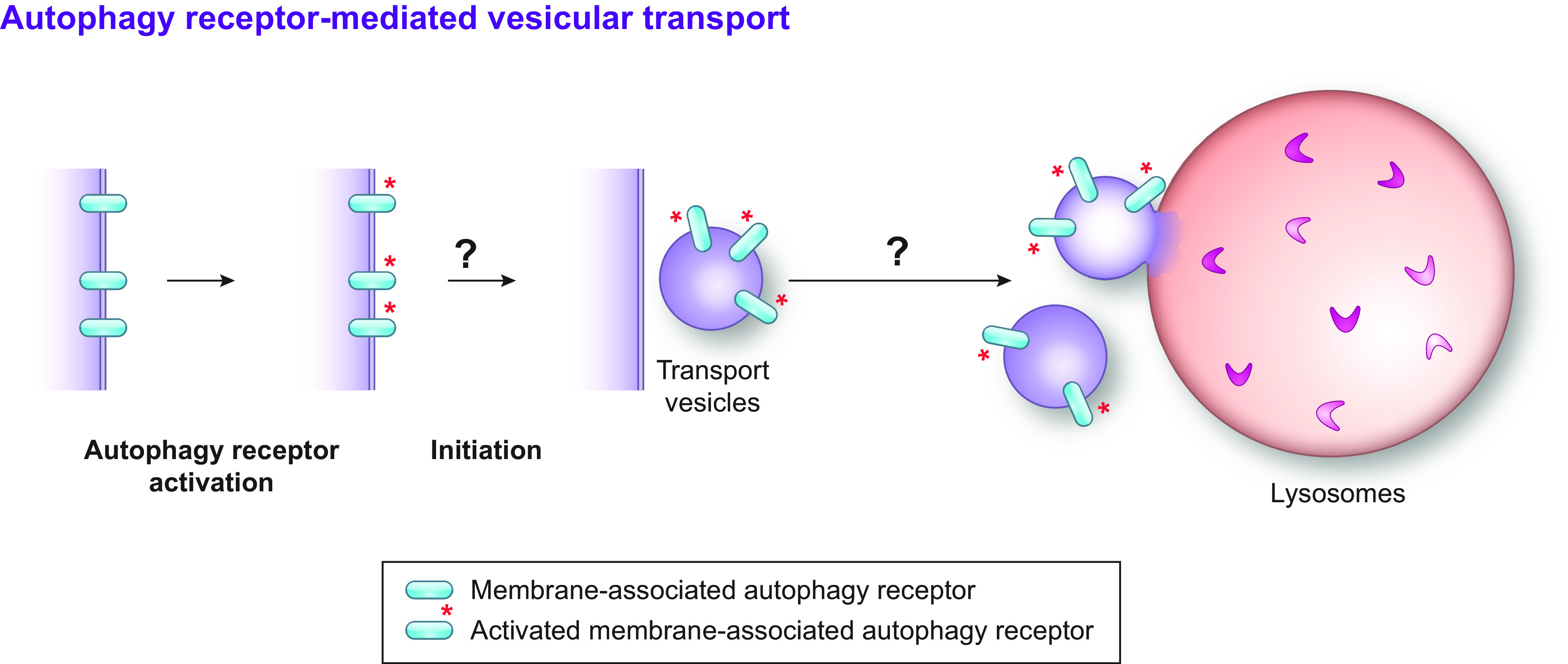
Autophagy receptor-mediated vesicular transport. Probably direct or indirect binding of the cargo to the autophagy receptor triggers the formation of a transport vesicle carrying both the cargo and the autophagy receptors. These transport vesicles subsequently directly fuse with compartments of the endolysosomal system. It remains unclear whether all the vesicular transport pathways involved in the degradation of a portions of a determined organelle always require autophagy receptors.

Selective autophagy pathways rely on the intervention of so-called autophagy receptors. These bind the cargo to be cleared on one hand and Atg8/LC3 proteins on the other hand (FIGURES 4–6). The binding between autophagy receptors and the Atg8/LC3 proteins relies on the short LC3-interacting region (LIR) motif (139, 140). Other motifs also exist, for example the Ub-interacting motif-like sequences that bind to an alternative site in the Atg8/LC3 proteins (141). These Ub-interacting motifs are degenerated sequences of ∼20 amino acids presented as an amphipathic α-helix and bind to a surface on the other side of Atg8/LC3 molecules relative to the sequence interacting with the LIR motifs (139). As such, autophagy receptors physically link the cargo to the forming autophagosomal membrane in the case of macro-autophagic events (FIGURE 4) (138, 142, 143). Moreover, autophagy receptors can facilitate the direct engulfment of cargoes by an endolysosomal compartment via micro-autophagy (FIGURE 5) or can be involved in autophagic processes, where portions of organelles fuse directly with the endolysosomal degradative compartments (FIGURE 6) (5, 66, 127). There are multiple types of autophagy receptors and various ways to classify them. They are often grouped based on how they recognize cargoes, i.e., Ub-mediated versus Ub-independent recognition. Whereas most of the Ub-binding autophagy receptors are soluble, those that are Ub independent are mainly membrane bound (139, 140, 142).

##### 2.1.2.2. cargo recognition mechanisms by autophagy receptors.

Soluble autophagy receptors (green in FIGURES 4 and 5) mediate selective macro- or micro-autophagy pathways by bridging cargoes with the membrane of either the autophagosomes or the degradative organelles (e.g., endosomes or lysosomes). For example, p62/SQSTM1, NBR1, Optineurin (OPTN), TAX1BP1, NDP52/CALCOCO2, and TOLLIP display LIR motifs and Ub-binding domains that associate with ubiquitylated cargoes to be removed from cells via macro-autophagy (FIGURE 4). Cargoes of ubiquitin-mediated macro-autophagy include bacteria (recognized by p62, OPTN, NDP52, and/or TAX1BP1), mitochondria (recognized by p62, OPTN, NDP52, and/or TAX1BP1), or protein aggregates (recognized by p62, NBR1, OPTN, TOLLIP, and/or TAXBP1) (138, 139, 142–148). In the latter case, recent work shows that for autophagic removal of ubiquitylated misfolded protein aggregates, p62-NBR1 heterooligomers drive and enhance the formation of Ub-positive protein condensates and that the NBR1 protein engages TAX1BP1, which in turn recruits FIP200, a core ATG protein, to promote autophagosome formation and cargo sequestration during macro-aggrephagy (149). The interaction between the autophagy receptors and Atg11/FIP200 appears to have the ultimate function to recruit and concentrate Atg1/ULK kinase complexes, leading to a spatial and temporal regulation of their activation that is essential to assemble the ATG machinery and initiate the formation of an autophagosome (150). Although not understood mechanistically yet, the docking of the Atg8/LC3 proteins to the autophagy receptors bound to the cargo could also play a relevant role in assembly and/or orchestration of the ATG machinery. These events probably mirror the supra-assembly of numerous Atg1/ULK kinase complexes via liquid phase separation that takes place during bulk macro-autophagy, which is key for the induction of autophagosome formation (77) (sect. 2.1.1.1). The central role of autophagy receptors in initiating macro- and possibly micro-autophagy, through FIP200 clustering, is also underlined during the LC3 lipidation-independent type of autophagy (151).

Membrane-associated autophagy receptors (blue in FIGURES 4 and 5), for instance mitophagy, pexophagy, nucleophagy, and a subset of ER-phagy receptors thoroughly discussed in the next sections, have been identified in yeast, plants, and Metazoa (5, 142, 144, 152). These intervene in the clearance of organelle portions, as in the case of mammalian mitophagy during red blood cell maturation and hypoxia, which depends on NIX/BNIP3L, BNIP3, and FUNDC1, three mitochondrial outer membrane proteins that mediate the selective sequestration of mitochondria into autophagosomes (153–156).

Whereas engagement of the Ub-dependent autophagy receptors is dictated by the ubiquitylation of the cargo destined for degradation, engagement of membrane-bound and of Ub-independent receptors is often achieved through phosphorylation. For instance, during organelle biogenesis, autophagy receptors such as Atg36 and Atg32 are present in an inactive, hypophosphorylated form in the membranes of yeast peroxisomes and mitochondria, respectively (140, 152). A change in growing conditions, which limits metabolism in these organelles, activates these receptors through their hyperphosphorylation, resulting in organelle clearance. Often, phosphorylations target the residues upstream of the LIR motifs, a principle that also applies to multiple Ub-dependent macro-autophagy receptors (140, 152). Acidic residues proximal to the LIR sequence increase affinity for Atg8/LC3 proteins, and a phosphorylated residue mimics an acidic amino acid, introducing two negative charges under physiological conditions. It must be noted that some times *1*) phosphorylations elsewhere in the autophagy receptors promote cross talk with downstream components of the ATG machinery (140, 152) and *2*) other posttranslational mechanisms regulating receptor activity exist, e.g., proteolytic processing or formation of homo- or heterooligomeric complexes (5, 140, 157).

##### 2.1.2.3. selective macro-autophagy.

In selective macro-autophagy, the binding of soluble autophagy receptors to the cargo or the activation of membrane-bound autophagy receptors triggers the recruitment and activation of the Atg1/ULK kinase complex, which promotes the formation of the phagophore adjacently to the structure selectively targeted for destruction, be it a macromolecule or a portion of an organelle (FIGURE 4, Cargo).

This was first demonstrated in yeast, in which autophagy receptors such as Atg19 engage Atg11, a subunit of the Atg1/ULK kinase complex (158, 159). As a matter of fact, Atg11 is essential for all the selective types of macro-autophagy in yeast (66). More recently, it has been shown that FIP200 is the mammalian functional counterpart of Atg11 and its interaction with soluble autophagy receptors initiates xenophagy, mitophagy, and aggrephagy (160–162). Engagement of Atg11 and FIP200 also takes place during yeast and mammalian macro-ER-phagy driven by the ER-phagy receptors CCPG1 (163), Atg39, and Atg40 (164, 165) (sects. 3.2.1.1.4 and 3.2.2.1), Atg39-mediated macro-nucleophagy in yeast (164, 166), and Nup159-mediated selective macro-autophagic degradation of nucleoporins and nuclear pore complexes (167, 168). The cross talk between the autophagy receptors and the Atg1/ULK kinase complex involves kinases such as Hrr25 in yeast and TBK1 in mammals, which phosphorylate the autophagy receptors, thereby increasing their binding affinity for the Atg8/LC3 proteins (161, 162, 169–171). The interactions between the autophagy receptors and Atg8/LC3 proteins probably also play an important role in the hermetic and exclusive sequestration of a cargo within an autophagosome (172). Upon activation of the Atg1/ULK kinase complex, the downstream steps that lead to the formation and consumption of an autophagosome appear to be identical to those taking place during bulk macro-autophagy (sect. 2.1.1.1).

##### 2.1.2.4. selective micro-autophagy.

Selective autophagy of macromolecules and portions of organelles such as nuclei, mitochondria, peroxisomes, and ER can also proceed in the absence of autophagosome involvement, when the cargo to be cleared from cells is directly engulfed by endolysosomal compartments through micro-autophagy (FIGURES 3 and 5) (5, 64, 66, 173–176).

Select examples are micro-mitophagy, which clears damaged portions of mitochondria in neurodegenerative disorders such as Huntington’s disease or upon nitrogen starvation in yeast (173, 177–179), micro-lipophagy, where lipid droplets are turned over by selective type II micro-autophagy in mammalian, plant, and yeast cells (113, 117–120), piecemeal micro-nucleophagy, which requires autophagy factors of the Ub-like conjugation system (180–182), and micro-pexophagy, which selectively removes peroxisomes (110, 183). Interestingly, pexophagy in methylotrophic yeast such as *Pichia pastoris* and *Hansenula polymorpha* proceeds by two different mechanisms depending on the induction conditions (113). When cultured in the presence of methanol, peroxisomes proliferate in these yeasts because this organelle contains the enzymes required for the metabolization of this carbon source. When methanol-grown cells are transferred to medium containing ethanol, excess peroxisomes are depredated via macro-pexophagy (113). When the same cells are transferred into a glucose-containing medium, however, superfluous peroxisomes are eliminated through a micro-pexophagy of type I (113). Finally, other examples of selective micro-autophagy are the ER stress- and misfolded protein-induced micro*-*ER-phagy of ER whorls, which are engulfed by yeast vacuoles (sect. 3.3.1.2.2) (64, 184, 185), and degradation of excess ER during recovery from ER stresses, which proceeds via piecemeal micro-ER-phagy (sect. 3.3.2.1) (45, 46, 186).

##### 2.1.2.5. direct fusion of select organelle portions with endolysosomal compartments.

A third pathway of selective autophagy consists in the turnover of organelle portions upon direct fusion of organelle-derived vesicles with the endolysosomal compartments (FIGURE 6).

This has been shown for mitophagy as triggered by oxidative stress (178, 187–191). In this catabolic transport pathway, oxidized components of the mitochondrial inner membrane and matrix are segregated and released from the healthy parts of the organelle in mitochondrial-derived vesicles (MDVs), a process regulated by the PTEN-induced putative kinase 1 (PINK1) and the E3 Ub ligase Parkin (178, 187–190, 192). MDVs engage the SNARE Syntaxin17, which eventually forms ternary complexes with the cytosolic SNAP29 and the late endosomal/lysosomal VAMP7 to mediate the fusion between MDVs and LAMP1-positive endolysosomal compartments (188). As such, the oxidative stress-induced, PINK1/Parkin-mediated turnover of mitochondria by MDVs differs from the PINK1/Parkin-regulated macro-mitophagy induced upon membrane depolarization because it does not require ATG5 and BECN1 (187). Similar pathways of selective organelle fragmentation and fusion of the organelle-derived vesicles with endolysosomal compartments to clear their content have been reported for ER-phagy as driven by the intraluminal accumulation of misfolded proteins (193–198). These latter pathways are thoroughly described in sect. 3.3.2.2.2.

## 3. AUTOPHAGY OF THE ER: ER-PHAGY

### 3.1. Opening Remarks

Autophagy pathways are triggered to gain nutrients, ensure physiological turnover of aging components, and protect cells from various types of stresses, including accumulation of cytotoxic material or pathogen attacks. Intuitively, autophagic events triggered upon shortage of nutrients do not require a high degree of selectivity. Under these conditions, cells need to rapidly generate metabolites required to fuel essential housekeeping functions and to ultimately guarantee survival. They do so by digesting cytoplasmic content, including organelles, upon activation of bulk autophagy and of a large spectrum of receptor-mediated autophagic programs. In other circumstances, however, the degradation of cell components must be regulated tightly. This occurs when the “eat-me signal” originates from an unwanted protein or protein complex, invading pathogens, or damaged portions of individual organelles such as mitochondria or the ER. These signals elicit very specific receptor-mediated autophagy responses that exclusively deliver a specific cargo to the endolysosomal/vacuolar district, preserving the other cellular components (sect. 2.1.2).

**FIGURE 7.**
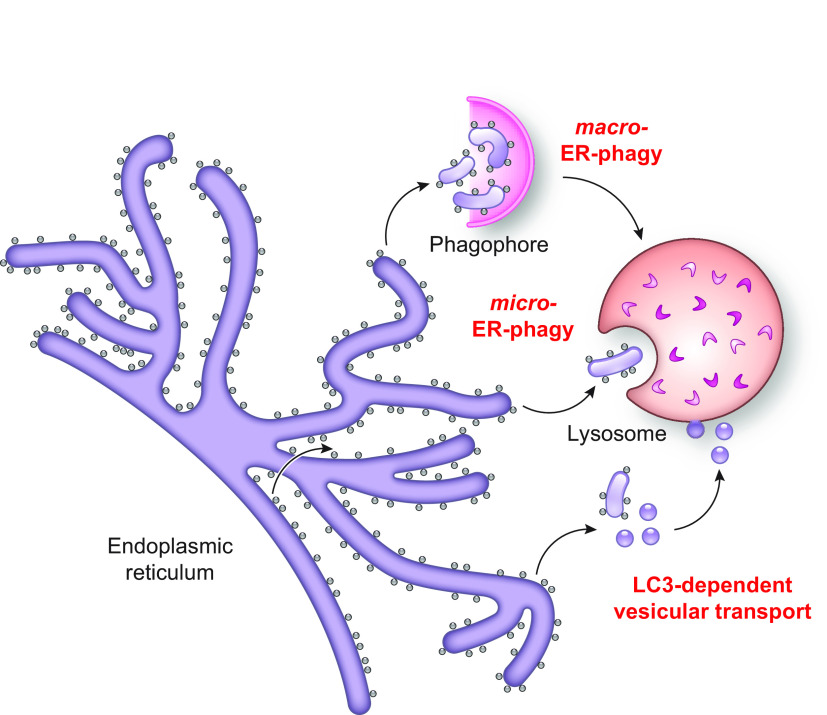
The 3 principal mechanisms of ER-phagy. ER fragments can be turned over by either macro- or micro-autophagy. Within these 2 pathways, ER fragmentation and sequestration into either autophagosomes or late endosomes/lysosomes/vacuoles are coordinated events. ER portions can also be directly transported to and fused with late endosomes and/or lysosomes/vacuoles. The different vesicular transport routes remain ill-characterized.

In this review, we report on ER-phagy responses and pathways that operate in mammalian, yeast, and plant cells to fragment and deliver ER portions to the endolysosomal/vacuolar compartments for clearance (FIGURE 7). These pathways drive rapid mobilization of proteins, membranes, and sugars to generate building blocks during nutrient restriction, regulate size, shape, and activity of the major biosynthetic organelle of nucleated cells, and control the quality of the cell’s proteome and lipidome by removing ER domains containing faulty gene products or aged and damaged constituents. Consistent with a relevant role in biology, ER-phagy and its regulators are hijacked by bacterial and viral pathogens to invade host cells and generate their progeny, and their misfunction may result in debilitating diseases. We here classify the ER-phagy responses according to the cues that initiate them, be these pleiotropic or ER-centric (sect. 3.3). Pleiotropic signals like nutrient deprivation, cellular stresses induced by chemical compounds, or light-dark cycles for plants activate the lysosomal turnover of the ER. However, this is part of a general cellular adaption that also involves the induction and inhibition of other pathways, including nonselective, bulk autophagy. ER-centric cues such as accumulation of misfolded polypeptides within the ER, recovery from ER stresses, ribosomes stalling on ER translocation channels, and infections activate the selective clearance of ER portions, leaving the cytoplasmic content and other organelles unaffected. We underline here that ER-phagy responses ensure the catabolic control of the ER content, shape, size, and functions and contribute with the anabolic unfolded protein responses to regulation of ER physiology in eukaryotic cells.

The anabolic unfolded protein responses are driven by activation of a few stress sensors embedded in the ER membrane. These sensors display large luminal domains that report on changes in ER homeostasis, to activate transcriptional and posttranscriptional responses (sect. 3.3.1.2.1). ER-phagy responses are driven by the concerted or individual activation of a much larger number of ER-phagy receptors. Several of them are embedded in the ER membrane, whereas others are recruited onto the ER membrane by specific signals. Most ER-phagy receptors lack luminal domains. We highlight below how they engage ER-resident adaptors (e.g., molecular chaperones) to mediate cargo segregation, membrane shaping and membrane shedding proteins, as well as cytosolic factors (e.g., members of the Atg8/LC3 protein family) to mediate delimitation and recognition of the ER subdomain that must be cleared, its detachment from the main ER body, and its delivery to the endolysosomal system for clearance (sect. 3.2). We also highlight that the pleiotropic or ER-centric signal-inducing ER-phagy responses determine the engagement of specific ER-phagy receptors and whether the ER portions targeted to destruction are captured by autophagosomes (macro*-*ER-phagy), engulfed by endosomes/lysosomes (micro*-*ER-phagy), or transported by LC3-dependent vesicular transport to endosomes/lysosomes with the intervention of specific core ATG proteins (FIGURE 7).

### 3.2. The ER-Phagy Receptors

**FIGURE 8.**
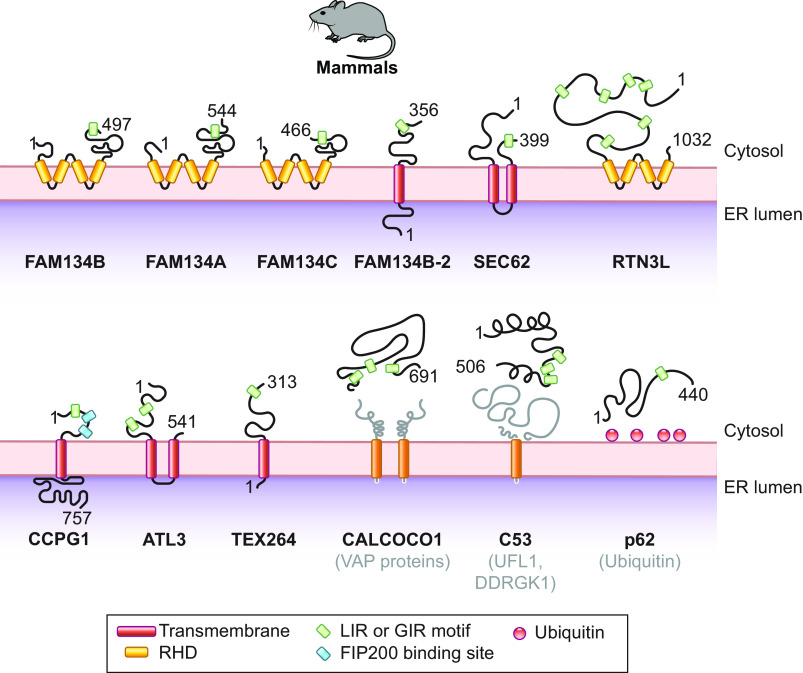
Schematic overview of the mammalian ER-phagy receptors. For simplicity, LIR or GABARAP interacting region (GIR) motifs indicate sequences that have been shown to bind members of the Atg8/LC3 protein family. Those sequences include conventional LIR motifs, nonconventional LIR motifs, and Ub-interacting motifs. Factors required to recruit soluble autophagy receptors to the ER, like VAP proteins, UFL1, DDRGK1, and Ub, are also shown. RHD, reticulon-homology domain. See glossary for other abbreviations.

**FIGURE 9.**
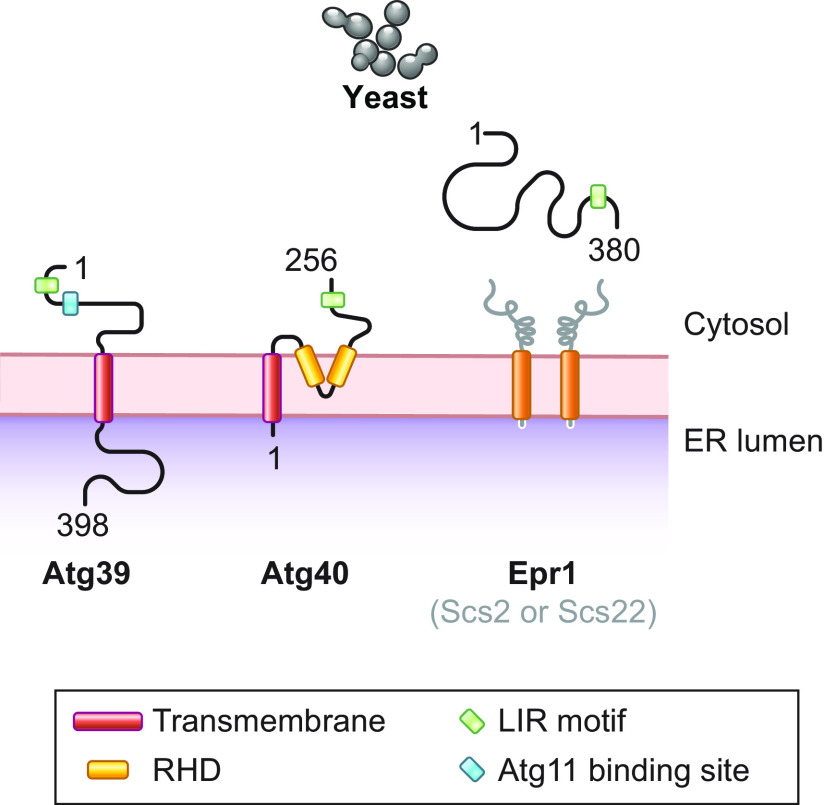
Schematic overview of the yeast ER-phagy receptors. Atg39 and Atg40 have only been studied in *S. cerevisiae*. Epr1 has been characterized in *S. pombe* and is not present in *S. cerevisiae*. Scs2 and Scs22 are yeast members of the VAP protein family that recruit soluble Epr1 to the ER. RHD, reticulon-homology domain. See glossary for other abbreviations.

**FIGURE 10.**
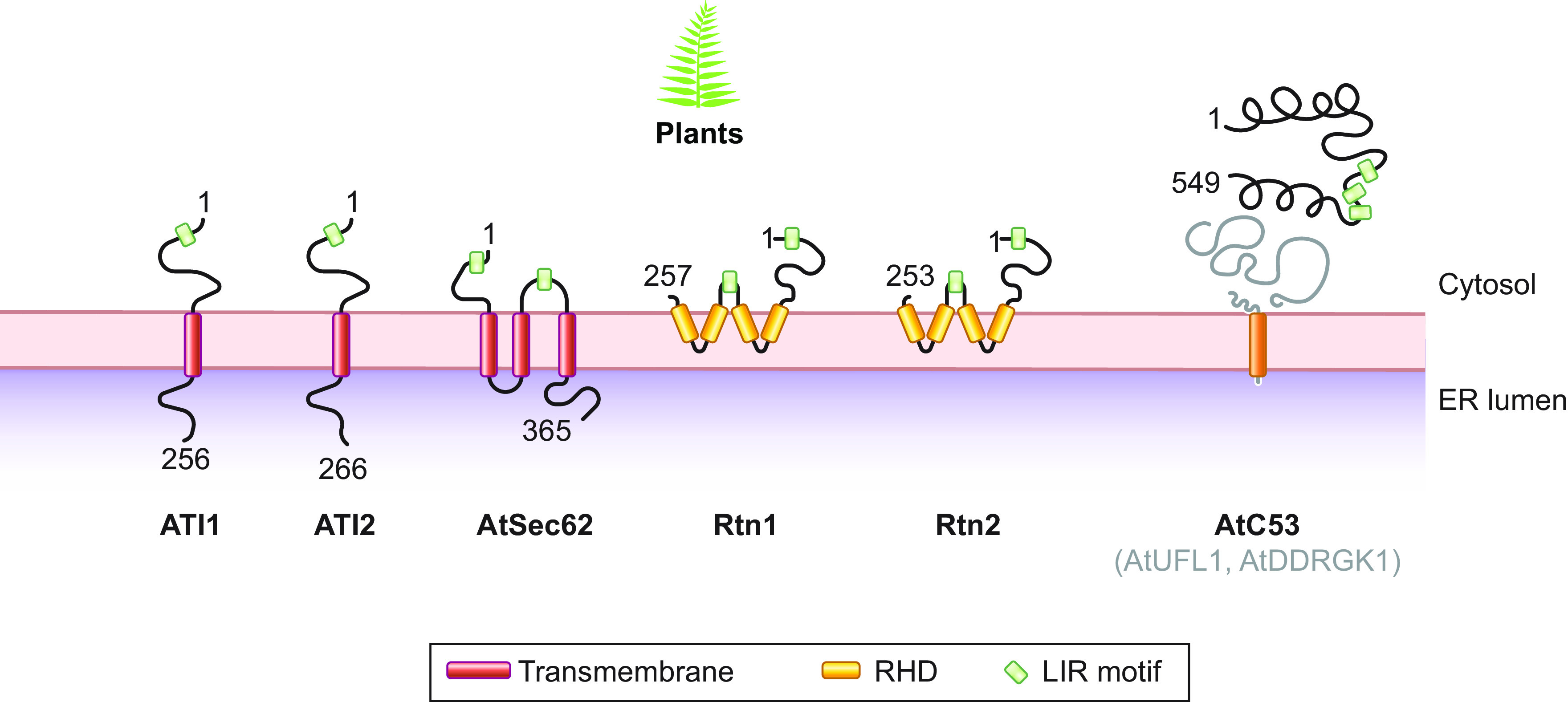
Schematic overview of the plant ER-phagy receptors. Membrane-bound (AtDDRGK1) and cytosolic (AtUFL1) factors required to recruit soluble autophagy receptors to the ER are shown. RHD, reticulon-homology domain. See glossary for other abbreviations.

The identification of ER-phagy receptors whose intracellular level and activity impact on fragmentation and delivery of ER portions into lysosomes/vacuoles for degradation has been a turning point for the study of ER-phagy. The first ER-phagy receptors to be characterized in mammalian, yeast, and plant cells are membrane-embedded ER proteins displaying one or more LIR motifs within their cytosolic domains. More recently, cytosolic Atg8/LC3-binding proteins, including well-established soluble autophagy receptors, have been shown to be recruited onto the ER membrane only upon ER-phagy activation and can operate as ER-phagy receptors. Upon activation, membrane-embedded and soluble ER-phagy receptors engage members of the Atg8/LC3 protein family and a variable cohort of luminal and cytosolic proteins and functional complexes that regulate self-digestion programs (FIGURES 8–10). As highlighted in the following subsections, the modularity of the supramolecular complexes that individual ER-phagy receptors can enter may explain their involvement in a vast array of ER-phagy programs and their versatility in participating in multiple and mechanistically distinct ER-phagy pathways.

#### 3.2.1. Mammalian ER-phagy receptors.

##### 3.2.1.1. membrane-bound er-phagy receptors.

###### 3.2.1.1.1. FAM134/RETREG protein family.

The protein family with sequence similarity 134 (FAM134) includes three conserved ER-resident proteins: FAM134B, FAM134A, and FAM134C (FIGURE 8) (199, 200). FAM134C is more abundantly expressed in cell lines and tissues, except for pancreas, which contains more FAM134B, and brain and testis, which express higher levels of FAM134A. FAM134B is enriched in ER sheets, whereas FAM134A and FAM134C have broader distribution within ER subdomains (199–202). The human proteins have 497 (FAM134B), 544 (FAM134A), and 466 (FAM134C) amino acids, respectively (FIGURE 8).

FAM134B was originally described as a tumor suppressor gene and named *JK1* (203). Subsequently, its role in long-term survival of nociceptive and autonomic ganglion neurons has also been reported (sect. 4) (204, 205). A yeast two-hybrid screen identified FAM134B as an interactor of Atg8/LC3 proteins. The subsequent observation of an ER swelling in cultured cells and in the cell body of pain-sensing neurons in mice upon deletion of *FAM134B* prompted the realization of a study that revealed an involvement of FAM134B in basal, housekeeping ER-phagy (200). Nutrient starvation substantially enhances FAM134B-driven macro-ER-phagy (200). More recently, FAM134A and FAM134C have also been involved in starvation-induced macro-ER-phagy (199, 206). This has led the HUGO gene nomenclature committee to introduce the names Reticulophagy Regulator1 (RETREG1), RETREG2, and RETREG3 for FAM134B, FAM134A, and FAM134C, respectively. In complex with the ER-lectin Calnexin (CNX), FAM134 proteins regulate macro-ER-phagy and LC3-driven vesicular transport pathways that ensure the lysosomal clearance of ER subdomains containing misfolded proteins that fail retrotranslocation across the ER membrane for proteasomal degradation (197–199, 207–209) (sect. 3.3.2.2.2). FAM134 proteins share an identical LIR motif at amino acid positions 454–460 (FAM134B), 491–497 (FAM134A), and 446–452 (FAM134C) (199) (FIGURE 8). The LIR motif of FAM134 proteins is located at the end of an intrinsically disordered region. This is a feature shared with other mammalian (RTN3L, CCPG1, SEC62, and TEX264), yeast (Atg39 and Atg40), and plant (ATI1 and ATI2) ER-phagy receptors (FIGURE 4).

The FAM134 proteins contain a reticulon-homology domain at amino acid residues 80–271 (FAM134B), 66–261 (FAM134A), and 53–244 (FAM134C), which is composed of two hydrophobic domains connected by a hydrophilic loop (FIGURE 8). Each hydrophobic domain forms a hairpin within the membrane bilayer without spanning it, promoting high membrane curvature and resulting in both the NH_2_ and COOH termini being exposed at the cytosolic leaflet of the ER membrane (200, 206, 210, 211). The reticulon-homology domain may contribute to membrane deformation, and clustering of FAM134 proteins may facilitate ER fragmentation (212). However, intervention of other factors, most notably the atlastin (ATL) GTPases (sect. 3.2.4), has been postulated to be required to vesiculate the ER portions that will eventually be delivered to the lysosomal/vacuolar district for clearance (5, 212–216).

FAM134B exists in two splicing variants. The full-length FAM134B is mainly expressed in the brain tissue and in glioblastoma cells, and its transcription is enhanced upon exposure to loperamide, a compound that induces ER stress and autophagy-mediated cell death (217–219). FAM134B-2 lacks the first 141 NH_2_-terminal residues, and it is mainly expressed in the liver and testis (204, 208, 220, 221) (FIGURE 8). The two variants of FAM134B are regulated in a cell- and tissue-specific manner by the transcription factor binding to IGHM enhancer 3 (TFE3) and the transcription factor EB (TFEB), two nutrient-responsive members of the microphthalmia/transcription factor E (MiT/TFE) family of transcription factors (222). *FAM134B-2* expression is enhanced by fasting through the C/EBPβ transcription factor, to regulate starvation-induced ER-phagy in the liver (208). In cultured HeLa cells, *FAM134B-2* expression occurs independently of C/EBPβ and is modulated by the transcription factors myocyte enhancer factor 2D (MEF2D), nuclear receptor subfamily 4 group A member 1 (NR4A1), BTB domain and CNC homolog 1 (BACH1), and zinc finger and BTB domain-containing 10 (ZBTB10) (223).

###### 3.2.1.1.2. SEC62.

SEC62 has been identified as the ortholog of the *S. cerevisiae* Sec62 subunit of the translocon complex, which controls translocation of newly synthesized proteins into the ER (224–227). Human SEC62 is an ubiquitously expressed, 399-amino acid, integral ER membrane protein that possesses two transmembrane segments and has only 16 residues within the ER lumen (FIGURE 8). Both the NH_2_ and the COOH terminus of the protein face the cytosol. The role of SEC62 in ER-phagy was first inferred by bioinformatic analyses revealing the presence of a putative LIR motif in its cytosolic COOH-terminal tail, at positions 363–366 (FIGURE 8), which is conserved in higher Eukarya (including plants; sect. 3.2.3.1) but not in *S. cerevisiae* (186). SEC62 lacks a reticulon-homology domain, but it interacts with putative reticulon-homology domain-containing ER-phagy receptors such as FAM134C (215), which may contribute to the membrane curvature required for ER fragmentation (206). The SEC62 LIR motif preferentially binds the lipidated form of Atg8/LC3 proteins (186), and, as in several other ER-phagy receptors, it is positioned at the end of a long intrinsically disordered domain (186, 228). SEC62 regulates the clearance of excess ER via micro-ER-phagy during cell recovery from ER stresses (45, 46, 186) (sect. 3.3.2.1). The treatment of cardiomyocytes exposed to chronic intermittent hypoxia with globular adiponectin induces *SEC62* expression and SEC62-driven ER-phagy to protect these cells from ER stress-induced death (229). The role of SEC62 in shaping ER size and function during recovery from ER stress is independent of its role in protein translocation. In fact, a functional LIR domain is dispensable for the latter activity (186). SEC62 has a functional ortholog, AtSec62, in plants (88, 230) (sect. 3.2.3.2). Notably, different cues determine the involvement of SEC62 in mechanistically distinct ER-phagy pathways. That is, SEC62 is involved in macro-ER-phagy, when activated by nutrient deprivation (sect. 3.3.1.1.2) (231), and in micro-ER-phagy, when activated during recovery from ER stresses (sect. 3.3.2.1) (45, 46). This parallels the versatility of FAM134 proteins, which regulate lysosomal ER turnover upon capture of ER fragments by double-membrane autophagosomes or upon direct fusion of ER-derived vesicles containing misfolded proteins with endolysosomes (197–199, 207–209). The mechanism by which ER-phagy receptors switch from one mode of ER-phagy to a different one remains unknown. Further studies will elucidate this and will reveal whether SEC62 and FAM134 functional versatility is an exception or is a feature shared with other ER-phagy receptors.

###### 3.2.1.1.3. RTN3L.

Together with members of the receptor expression-enhancing protein (REEP) family, reticulon (RTN) proteins are necessary and sufficient to generate membrane tubules and to fuse them, with intervention of the membrane-anchored GTPases of the ATL protein family, to form the intricated network of tubules and sheets composing the ER (232–235). The RTN protein family comprises four proteins, RTN1, RTN2, RTN3, and RTN4, which are expressed in several splicing variants. All have conserved reticulon-homology domain and COOH-terminal domains, but their NH_2_ termini, which determine the interacting partners, are highly variable. RTN proteins are enriched in tubular ER, and overexpression analyses in cells subjected to nutrient deprivation revealed that only RTN3L, the large isoform of RTN3, induces ER fragmentation and colocalization between ER and Atg8/LC3 proteins and promotes starvation-induced, lysosomal clearance of ER tubuli (236). RTN3L is a 1,032-amino acid polypeptide (FIGURE 8). It possesses two reticulon-homology domains at the protein’s COOH terminus, six LIR motifs distributed within the 800 residues that are part of its cytosolic NH_2_-terminal intrinsically disordered domain, and no significant portion facing the ER lumen (236) (FIGURE 8). Comparison of the RTN protein interactome revealed that only RTN3L forms complexes with GABARAP and GABARAP-L1, with proteins linked to vesicular transport including SEC22B, SEC23A, SEC23B, and SEC24C, and with proteins regulating ER membrane contacts with other organelles such as VAPA and VAPB (236). A recent report has shown that the LIR motifs of RTN3L contribute to endosomal maturation and cargo sorting by establishing membrane contact sites between the ER and endosomes upon association with phenylalanine-serine-valine motifs in RAB9A (237). RTN3L has two functional orthologs in plants, Rtn1 and Rtn2 (238) (sect. 3.2.3.3).

###### 3.2.1.1.4. CCPG1.

Cell cycle progression gene 1 (CCPG1) is a vertebrate-specific protein that reestablishes cell cycle progression in *S. cerevisiae* arrested in the G1 phase when overexpressed (239). A role of CCPG1 as a scaffold protein ensuring assembly and controlling specificity of various signaling complexes has also been reported (240). The relevance of these two findings for vertebrate physiology remains unclear. CCPG1 is a single type II transmembrane ER protein of 757 amino acids (FIGURE 8), which is expressed in several isoforms. It is abundantly present in pancreas, stomach, kidney, and liver (163). The expression of *CCPG1* is regulated by the transcription factor basic helix-loop-helix family member a15 (BHLHA15/MIST1) (241), whose expression depends on the ER to nucleus signaling 1 (ERN1/IRE1)/X-box binding protein 1 (XBP1) branch of the unfolded protein responses in professional secretory cells of various organs, including the pancreas (242, 243). Consistently, *CCPG1* is an ER stress-induced gene, whose transcription is enhanced in cells exposed to thapsigargin, tunicamycin, or dithiothreitol (163). *CCPG1* is among the top 50 genes that are upregulated during B cell-to-highly secreting plasma cell differentiation. This latter process is also controlled by XBP1 and requires the potentiation of the cellular protein disposal machineries (244–247).

CCPG1 associates with Atg8/LC3 proteins via a LIR motif at amino acid positions 14–17 and with FIP200 via two FIP200-interacting regions at positions 18–29 and 101–110, which are in its cytosolic NH_2_-terminal intrinsically disordered domain (FIGURE 8). In contrast to the vast majority of membrane-associated ER-phagy receptors, CCPG1 displays a long luminal domain comprising 518 COOH-terminal residues (163) (FIGURE 8). The long luminal domain and the association with FIP200 are features that CCPG1 shares with the *S. cerevisiae* ER-phagy receptor Atg39 (FIGURE 9, sect. 3.2.2.1). Interactions with Atg8/LC3 proteins and FIP200 are required for lysosomal clearance of ER structures containing CCPG1, which are induced by either nutrient deprivation or ER stress (163). As such, CCPG1 contributes with many other ER-phagy receptors to starvation-induced ER-phagy (163, 200, 208, 214, 228, 231, 236, 248–250) (sect. 3.3.1.1.2). Together with SEC62, TEX264, ATL, and all soluble ER-phagy receptors, CCPG1 is part of the ER-phagy receptor subset lacking a reticulon-homology domain. These ER-phagy receptors are likely to rely on interactions with membrane shaping proteins (sect. 3.2.4), including RTN and/or ATL, to fragment the ER. CCPG1’s role in starvation-induced ER-phagy and removal of misfolded polypeptides from the ER is highlighted by the accumulation of insoluble acinar secretory enzymes and ER swelling in hypomorphic *Ccpg1* mice (163).

###### 3.2.1.1.5. Atlastin GTPases.

Membrane-anchored Atlastin (ATL) GTPases form complexes with ER-shaping proteins, including RTN and REEP proteins, which promote the membrane fusion events that lead to the formation of the three-way junctions involved in the generation of the lacelike ER structure (232–235). Mammals express ATL1 in the *cis*-Golgi of cells of the nervous system and ATL2 and ATL3, which can form heterodimers, in the ER of other tissues (213, 251, 252). A model predicting that, similarly to RTN and REEP proteins, the transmembrane domains of ATL proteins do not span the ER membrane lipid bilayer and contribute to enhancing membrane curvature remains to be confirmed (253). In particular, ATLs localized to adjacent membranes form dimers in *trans* via their cytosolic GTPase domains, and the subsequent GTP hydrolysis pulls the membranes in close proximity and fuses them (234, 235).

The first evidence of ATL proteins being involved in ER-phagy emerged with the observations that *ATL2* deletion substantially impairs starvation-induced clearance of ER sheets and prevents ER delivery into lysosomes upon overexpression of FAM134B (213). This phenotype can be rescued by the single overexpression of each one of three ATL proteins, and it requires both formation of homo- or heterodimers and their GTPase activity (213). The authors of this latter study proposed a model in which ATL2 induces shedding of FAM134B-containing ER subdomains, based on the reported activity of ATL1 in ER-derived vesicles formation (254). Subsequent work that recapitulated the involvement of ATL2 as a putative downstream regulator of FAM134B in the vesiculation of ER sheets also reported that levels of ATL3, which is enriched in ER tubuli, increase significantly in the early phases of starvation (it remains unclear whether this relies on transcriptional, translational, or posttranslational control) and that ATL3 plays a direct role as an ER-phagy receptor in starvation-induced ER-phagy of tubular ER (214). In contrast to ATL2, which does not associate with Atg8/LC3 proteins, ATL3 selectively binds GABARAP (but not LC3) via two GABARAP interacting motifs (GIMs) at amino acid positions 192–195 and 390–393, which are specific for this subset of Atg8/LC3 proteins (214) (FIGURE 8). The first of these motifs is conserved in ATL1, at position 196–199. However, although ATL1 association with GABARAP has been confirmed, a role of ATL1 as an ER-phagy receptor awaits experimental evidence (214). Notably, ATL2 and ATL3 coordinate the assembly of the ULK kinase complex adjacently to the ER during formation of autophagosomes (255). This raises the interesting possibility that ATL proteins coordinate cargo recruitment and ATG machinery assembly to guarantee the specific sequestration of determined ER subdomains, as observed in other selective types of autophagy (sect. 2.1.2).

###### 3.2.1.1.6. TEX264.

Testis expressed protein 264 (TEX264) was first mentioned in the literature as a database-deposited protein-encoding open reading frame (256). The gene is transiently expressed in several tissues, e.g., primary spermatocytes during spermatogenesis (257). It has been identified as an ER-resident protein cleared from cells in an ATG5- and ATG7-dependent manner (228, 248). TEX264 is a single type I transmembrane ER protein of 313 amino acids, only 6 of which protrude in the ER lumen (FIGURE 8). TEX264 localizes in three-way ER tubule junctions and moves in punctuate structures positive for Atg8/LC3 proteins upon nutrient starvation (228, 248). Association with lipidated Atg8/LC3 proteins depends on a LIR motif conserved in Metazoa, located within its COOH-terminal intrinsically disordered domain, at amino acid position 275–278 (FIGURE 8). This long, intrinsically disordered domain might serve to bridge the membrane of the rough ER, in which ribosomes protrude for ∼20 nm, with the LC3/Atg8 molecules anchored onto the membrane of the phagophores or endolysosomal compartments (197, 228, 258).

Proximity biotinylation-mass spectrometry revealed a number of proteins contiguous to TEX264 at steady state, including abundant ER membrane proteins like CNX and thioredoxin related transmembrane protein 1 (TMX1) (sect. 3.2.4), autophagy regulators such as ATG14, WIPI2, and RAB7A, and macro-autophagy receptors including p62, NDP52, and TAX1BP1 (248). Induction of ER-phagy by nutrient starvation and concomitant inhibition of lysosomal degradation by treatment with bafilomycin A1 increased the amount and variety of ER proteins in the proximity of TEX264 molecules, underscoring its ER-phagy receptor function (248). Thus, like most of the ER-phagy receptors studied so far, TEX264 contributes to nutrient starvation-induced turnover of the mammalian ER (228, 231, 248). Like FAM134B, TEX264 expression is transcriptionally enhanced upon exposure to loperamide, a compound that induces ER stress and autophagy-mediated cell death (217–219).

###### 3.2.1.1.7. STING.

Cyclic di-adenosine monophosphate (cyclic di-AMP) produced by Gram-positive bacteria binds to the ER tetraspannin stimulator of interferon response cGAMP interactor 1 (STING) (259). This triggers ER stress and activates autophagy responses, including ER-phagy, upon mTOR inhibition (260). ER-phagy induction relocalizes STING (261) from the ER to the phagophore membrane, from where STING induces an interferon I response (260). These pathogen-induced ER-phagy responses relieve the bacteria-induced ER stress by removing ER membranes and proteins, including STING itself. Recently, it has been shown that STING contains three LIR motifs at residues 167–170, 199–202, and 245–248 and directly interacts with Atg8/LC3 proteins (259, 262). For this reason, STING is tentatively included among the membrane-bound ER-phagy receptors. Interestingly, STING activation induces LC3 lipidation on single-membrane vesicles or organelles, which depends on ATG16L1, but not on components of the ATG machinery such as ULK1 or ULK2, VPS34, BCN1, and ATG9A that intervene in conventional LC3 lipidation during autophagosome biogenesis (263). It has been postulated that STING-induced LC3 lipidation and subsequent fusion of STING-containing ER-derived vesicles with degradative compartments of the endolysosomal system ensures the elimination of invading pathogens or their intracellular trafficking through the endocytic/secretory pathways (263).

##### 3.2.1.2. cytosolic er-phagy receptors.

###### 3.2.1.2.1. CALCOCO1.

CALCOCO1/CoCoA was first described as a nuclear receptor coactivator (264) whose expression may correlate with tumorigenic activity in breast cancer (265). It is a paralog of the soluble autophagy receptors NDP52/CALCOCO2 and TAXBP1/CALCOCO3 (249, 250, 266–268). CALCOCO1 is a 691-residue cytosolic protein that, with NDP52 and TAXBP1, shares a SKICH domain (residues 15–119), which has been proposed to facilitate protein:protein or protein:lipid associations (249, 250) (FIGURE 8). The two studies that have investigated the role of CALCOCO1 in macro-ER-phagy (249, 250) have reached different conclusions. One study established that a canonical (residues 46–53) and an atypical (residue 141–143) LIR motif regulate the function of CALCOCO1 in macro-autophagy (250). The other showed that the atypical LIR motif binds GABARAP proteins cooperatively with a Ub-interacting motif at amino acid position 615–653 (249). This latter study did not observe binding between canonical LIR motif (residues 46–53) and LC3/Atg8 proteins (249). Like all other soluble ER-phagy receptors (sects. 3.2.1.2, 3.2.2.2, and 3.2.3.4), the role of CALCOCO1 in ER-phagy relies on its recruitment onto the ER membrane, which is elicited by nutrient deprivation and requires binding to VAPA or VAPB (FIGURE 8) (249). VAP proteins are integral membrane ER proteins deputed to the formation of membrane contact sites with other organelles by binding proteins bearing the VAP-interacting motif FFAT, which is composed of two phenylalanine residues (FF) in an acidic tract (AT) (269). Sequence and mutation analyses identified the FFFSTQD sequence in CALCOCO1 at residues 680–686 and a flanking acid tract as the potential FFAT-like motif required for its interaction with VAP proteins (249).

More recently, CALCOCO1 and TAXBP1 have been involved in the lysosomal turnover of Golgi subdomains. This is driven by association to the ankyrin repeat domain of the Golgi-resident palmitoyltransferases zinc finger DHHC-type palmitoyltransferase 17 (ZDHHC17) and ZDHHC13, which is ensured by short ZDHHC-AR-binding motif spanning residues 574–579 of human CALCOCO1 and residues 673–678 of TAXBP1 (249, 270, 271). As a note, TAXBP1 lacks the VAP-interacting domain that recruits CALCOCO1 onto the ER, and this may explain why it is not involved in ER-phagy.

###### 3.2.1.2.2. C53.

C53/CDK5rap3/LZAP is a 506-amino acid cytosolic protein (FIGURE 8) expressed in all tissues, which is conserved in vertebrates, invertebrates, and plants but not in yeast (272–274). The role of C53 in regulation of cell cycle, cell survival, cell adherence, tumorigenesis, and metastasis relies on interactions with several proteins including tumor suppressors and oncogenes (273–276). For its role in ER-phagy, C53 interacts with the ER-associated complex formed by the UFM1-specific E3 ligase (UFL1) and its DDRGK domain containing 1 (DDRGK1) adaptor (FIGURE 8) to protect cells from ER stress (277–281). The interaction between C53, UFL1, and DDRGK1 changes the UFMylation profile of liver proteins and controls ER homeostasis in the regenerating liver (282, 283). UFMylation is the covalent attachment of the Ub-like protein ubiquitin fold modifier 1 (UFM1) to target proteins via the sequential action of the E1-like activating enzyme ubiquitin-like modifier activating enzyme 5 (UBA5) and the E2-like ubiquitin-fold modifier-conjugating enzyme 1 (UFC1). The modification can affect stability, spatial conformation, and ability of the target protein to interact with partners (284). C53 (and its plant orthologs AtC53 and MpC53, sect. 3.2.3.4) interacts with Atg8/LC3 proteins via three conserved LIR motifs at positions 267–270, 292–295, and 310–313 in the human protein and with Atg11/FIP200. The finding that a conditional knockout of C53 in mouse results in accumulation of ER proteins led to the hypothesis of an involvement of both C53 and the UFMylation machinery in ER-phagy (278, 282). C53 is dispensable when ER-phagy is triggered by nutrient deprivation, although the UFMylation machinery still plays a relevant role (231). As described in sect. 3.3.2.3, C53 enters into play when ER-phagy is induced by the clogging of translocons with nascent proteins emerging from ribosomes stalled on faulty mRNA (278).

##### 3.2.1.3. other autophagy receptors involved in er-phagy.

An increasing number of autophagy receptors mediate lysosomal turnover of multiple cargoes, including the ER (5, 66, 142, 271). The most-studied member of the family of multitask autophagy receptors, which also includes NBR1, OPTN, NDP52, and TAXBP1, is p62/SQSTM1 (FIGURE 8), which promotes the autophagic clearance of ubiquitylated cargos, including pathogens and organelles such as peroxisomes, mitochondria, and the ER. The function of p62 as ER-phagy receptor is exerted by recognizing ER membrane proteins that are ubiquitylated after termination of pharmacological treatments in liver cells (285) and in cells experiencing an ER stress induced by chemical compounds perturbing calcium homeostasis, *N*-glycosylation, or causing accumulation of misfolded proteins upon proteasomal inhibition (286, 287). In this latter context, the E3 ligase tripartite motif containing 13 (TRIM13) ubiquitylates itself, triggering the subsequent recruitment of p62 onto the ER membrane. The binding of the TRIM13-p62 complex with proteins arginylated at their NH_2_ terminus promotes the formation of TRIM13-p62 polymers that initiate ER-phagy (286, 287).

NBR1 and OPTN are two other cytosolic Ub-binding autophagy receptors. They have been implicated in the control of ER stress magnitude by turnover of the ER stress transducer IRE1 (136). Since IRE1 is an integral ER membrane protein, this observation may suggest that they are involved in an ER-phagy pathway.

#### 3.2.2. Yeast ER-phagy receptors.

##### 3.2.2.1. atg39 and atg40.

*S. cerevisiae* Atg39 localizes in the perinuclear ER membrane, an extension of the ER that generates the outer and inner nuclear membrane. Atg39 is a type II transmembrane protein of 398 residues (FIGURE 9) that, together with Atg40, has been identified as an Atg8 interactor upon affinity purification coupled with protein mass spectrometry (164). Like CCPG1 (163), the long NH_2_-terminal cytosolic intrinsically disordered domain of Atg39 binds both Atg8, via a LIR motif at residues 5–13, and Atg11, via a sequence at residues 52–59 (FIGURE 9) (164). Uncommon among ER-phagy receptors, but again like CCPG1, the 234 amino acid residues at the COOH terminus of Atg39 protrude into the lumen of the ER and in the perinuclear space. It has been hypothesized that Atg39 contributes to the formation of nuclear envelope-derived double-membrane vesicles, in which the external membrane corresponds to the outer nuclear membrane and the internal membrane corresponds to the inner nuclear membrane. These are eventually sequestered by autophagosomes and delivered into the vacuole (288). As such, Atg39 should be considered both an ER-phagy and a nucleophagy receptor (164). Recent data defining the role of Atg39 in nucleophagy reveal that the COOH-terminal region of the protein contains short amphipathic helices that may tether the outer and the inner nuclear membrane directly (166) or via interaction with inner nuclear membrane proteins (289). This would promote the vesiculation of the nuclear envelope and its capture by autophagosomes. Notably, the ER-phagy and nucleophagy pathways driven by Atg39 are required for cell survival upon nitrogen starvation (164).

Atg40 is a 256-amino acid protein localized to the cortical and cytosolic ER, with a transmembrane domain at the NH_2_ terminus, a reticulon-homology domain, and a long cytosolic COOH-terminal intrinsically disordered region, displaying a LIR motif at residues 239–247 (FIGURE 9). The overexpression of Atg40 complements the deletion of *S. cerevisiae* reticulons Rtn1, Rtn2, and Yop1, thus indicating that Atg40 is a RTN-like protein that alone may generate high curvature of the ER membrane (84). On the same line, Rtn1 and Yop1 fused with the LIR motif of Atg40 at their COOH termini restore starvation-induced ER-phagy in cells lacking Atg40 (84). Notably, the interaction of Atg40 with Atg8 is stabilized by a short helix positioned after the LIR motif in the primary protein structure, which is conserved in mammalian receptors such as FAM134B, RTN3L, and SEC62 (84). Finally, deletion of *ATG40* only partially inhibits starvation-induced ER-phagy. To achieve a more complete inactivation of the ER turnover in the vacuole, *ATG39* also has to be knocked out (164). Atg40 has also been involved in removing ER subdomains containing misfolded polypeptides (sect. 3.3.2.2.2.1).

Expression of Atg39 and Atg40 is induced by nutritional or pharmacological mTORC1 inactivation, chemically induced ER stresses, or accumulation of misfolded proteins in the ER. Induction does not depend on Xbp1-mediated unfolded protein responses (290). It rather relies on derepression of the *ATG39* and *ATG40* gene transcription. For Atg39, this requires phosphorylation of the transcriptional repressors Mig1 and Mig2 by the AMP-activated protein kinase Snf1, which leads to their release from the gene promoter (290). The upregulation of Atg39 in yeast cells lacking Snf1 has recently led to the identification of the transcription factors Msn2 and Msn4 as inducers of Atg39 expression (291). For Atg40, transcriptional derepression is controlled through inactivation of the components of histone deacetylase complex Rpd3 and Pho23 (292).

##### 3.2.2.2. epr1/mug185.

So far, only one soluble ER-phagy receptor, Epr1, has been reported (in *S. pombe*) (293) (FIGURE 9). Epr1 has been identified upon affinity purification coupled with protein mass spectrometry aimed at identifying Atg8 interactors. Like the mammalian ER-phagy receptor CALCOCO1, recruitment of Epr1 onto the ER membrane upon chemically induced ER stresses is mediated by its binding to the two yeast members of the VAP protein family, Scs2 and Scs22 (293). Epr1 translation is enhanced by the activation of the ER stress transducer Ire1 upon exposure to dithiothreitol, through a mechanism that remains to be elucidated.

#### 3.2.3. Plant ER-phagy receptors.

##### 3.2.3.1. ati1 and ati2.

The *Arabidopsis thaliana* ER-resident Atg8-interacting proteins ATI1 and ATI2 were the first ER-phagy receptors to be reported in the literature (294). They are type II transmembrane proteins of 256 and 266 residues, respectively (FIGURE 10), which were shown to interact with Atg8/LC3 proteins by yeast split Ub assay and Atg8f and Atg8h as baits (294, 295). Consistently, they display a LIR motif within their long cytosolic NH_2_-terminal intrinsically disordered domain (258, 296) (FIGURE 10). Dark-induced carbon starvation triggers ATI1 and ATI2 association with Atg8f and segregation in spherical ER bodies, which are eventually transported into the vacuole for clearance via direct ER-to-vacuole trafficking (294, 297, 298).

These ER bodies, which colocalize with both ATI1 and ATI2, accumulate in the vacuole when cells are exposed to concanamycin A. This antibiotic raises the vacuolar pH by inhibiting the vacuolar ATPase inactivating the proteolytic enzymes (299) and proves the involvement of these two receptors in ER-phagy (296). Dark-induced ER-phagy also regulates the turnover of the ER-localized membrane steroid binding protein 1 (300). ATI1 and ATI2 have no homologs in yeast and higher Eukarya.

##### 3.2.3.2. atsec62.

In *A. thaliana*, ER-phagy is triggered by ER stress induced by the perturbation of the redox homeostasis, inhibition of protein *N*-glycosylation (301), or luminal expression of misfolded proteins (302). ER stress-induced ER-phagy relies on the activation of the membrane stress sensor AtIre1B, whose RNase activity is required for clearance of transcripts encoding for a variety of autophagy repressors (303). ER stress-induced ER-phagy is mediated by the SEC62 ortholog AtSec62, which colocalizes and interacts with Atg8e (88, 230). In plants, AtSec62 is a tri-spanning ER membrane protein of 365 residues required for plant development and protecting cells from ER stress (FIGURE 10). Its COOH terminus faces the ER lumen, and the NH_2_ terminus is exposed to the cytoplasm and displays the Atg8e interacting domain (FIGURE 10). A second LIR motif is located between transmembrane domains 2 and 3 (FIGURE 10) (88).

##### 3.2.3.3. rtn1 and rtn2.

Maize Rtn1 and Rtn2 are 257- and 253-amino acid RTN proteins (FIGURE 10) present in the ER of starchy endosperm and aleurone cells, two seed tissues, during the storage of compounds that will eventually be metabolized during germination (238). These two proteins have 72- and 68-amino acid NH_2_-terminal cytosolic extensions, respectively, that contain a LIR motif (FIGURE 10). Their association with Atg8/LC3 proteins has been shown with the yeast split Ub assay and Atg8a as the bait. Other LIR motifs are in the cytosolic loop between intramembrane domains 2 and 3 and in the protein’s intramembrane domains (the latter not shown in FIGURE 10). Rtn1 and Rtn2 expression is induced by ER stress and promotes the vacuolar turnover of the ER, as shown by the accumulation of ER within the vacuole in cells treated with concanamycin A (238). Like mammalian ER-phagy receptors such as the members of the FAM134 protein family and TEX264 (197–200, 248), plant Rtn1 and Rtn2 interact with luminal ER chaperones, namely CNX, binding immunoglobulin protein (BiP), and protein disulfide isomerase (PDI) (238). Their interaction with these chaperones is likely to mediate association of cargo proteins that need to be cleared or to allow the transduction of intrinsic signals that elicit turnover of select ER subdomains (5). The overexpression of Rtn1 and Rtn2 changes the ER architecture, but it is not sufficient to drive ER fragmentation. As in Metazoa and yeast, it is likely that ER fragmentation requires the intervention of other proteins that can operate membrane scission (sect. 3.2.4) (5, 213, 214). Candidate proteins for this function in plants are members of the Root Hair Defective 3 (reticulon-homology domain3) protein family (238). These are putative GTPases whose intracellular level is reportedly regulated by the Lunapark proteins Lnp1 and Lnp2 (304). Notably, the yeast ortholog of Lnp1 has been involved in starvation-induced, Atg40-driven macro-ER-phagy (165).

##### 3.2.3.4. atc53 and mpc53.

The soluble *A. thaliana* and *Marchantia polymorpha* proteins Atc53 and Mpc53 are the plant orthologs of mammalian C53. They associate with Atg8/LC3 protein isoforms via three shuffled versions of the canonical LIR motif and with Atg11/FIP200 (FIGURE 10). The association of the C53 orthologs with Atg8 proteins and Atg11 is not triggered upon carbon starvation, which is often used to investigate bulk autophagy in plants. Rather, it is induced by chemicals such as cyclopiazonic acid and tunicamycin that elicit ER stresses, phosphate starvation, and protein translation inhibitors that may block nascent polypeptides within the translocon (278, 305). In these contexts, the colocalization of Atc53 and Mpc53 with Atg proteins is increased upon cell treatment with concanamycin A. Intriguingly, the puncta positive for both AtC53 and Atg8, which probably represent autophagosomes, do not colocalize with the ER lumen marker GFP-HDEL but rather with UFL1 and DDRGK1. This colocalization is enhanced upon ER stresses, which also induce the transport of the complex formed by these two proteins within the vacuolar lumen (278).

#### 3.2.4. ER-phagy receptors: demarcation and fragmentation of ER portions for lysosomal clearance.

The roles of ER-phagy receptors in the ER-phagy pathways beyond cargo recognition and coordination of the sequestration machinery are not fully understood. Those displaying reticulon-homology domains (FIGURES 8–10) certainly play a role in the deformation of the ER membrane that precedes the poorly understood scission of subdomains of this organelle, which generates the ER fragments that will eventually be cleared in the compartments of the endolysosomal system. The demarcation and fragmentation of ER portions is an essential and poorly defined part of the ER-phagy pathways. As stated above, most ER-phagy receptors have very short amino acid stretches that protrude into the ER lumen (the soluble receptors nothing at all). Most of them rely on the association with other ER proteins to sense perturbations in the ER lumen and/or demark the membrane portions that encode an eat-me signal. The best-known case is the association of FAM134 protein family members with the lectin chaperone CNX (197, 198, 200), whose persistent binding to misfolded proteins can segregate them in ER subdomains that eventually vesiculate and are degraded with their content via ER-to-lysosome-associated degradation pathways (197, 198, 207) (sect. 3.3.2.2.2). Associations between TEX264, CNX, and the ER membrane-bound oxidoreductase TMX1 and of plant Rtn1 and Rtn2 with CNX, BiP, and PDI have also been reported, but their relevance in selecting cargo and/or delimiting the ER subdomains to be removed remains to be understood (238, 248, 306). Moreover, the transmembrane ER protein Progesterone Receptor Membrane Component 1 (PGRMC1) interacts with RTN3 proteins and mediates the clearance of ER subdomains containing a subset of misfolded clients during RTN3L-mediated ER-phagy (307) (see also sect. 3.3.2.2.2.4).

How ER portions are fragmented from the main ER body is still ill-defined. Local concentration of membrane proteins may, under some experimental conditions, promote membrane scission (308, 309) or generate lipoprotein particles, respectively (310). Computational molecular dynamics simulations have revealed that clustering of multiple reticulon-homology domains that are present in some but not all ER-phagy receptors induces formation of ER membrane buds (212, 216). The monolayer or bilayer structural perturbation introduced by clustering of reticulon-homology domain-containing proteins is not sufficient to fragment the ER but is expected to facilitate it. Clustering of ER-phagy receptors such as Atg40 (84, 311) and TEX264 (248) is induced by association with Atg8/LC3, which forms oligomers upon lipidation on the phagophore in cells exposed to pleiotropic signals (84, 311). Clusters of ER-phagy receptors are stabilized by a short, conserved, helical region at the COOH terminus of the LIR motifs of Atg40, SEC62, FAM134B, and RTN3L (84, 311). For ER-phagy receptors like mammalian CCPG1 and yeast Atg39, which bind FIP200 and Atg11, respectively, the involvement of foci of lipidated LC3 in selective autophagy is bypassed by the formation of FIP200 and Atg11 clusters (151). Like the RTN-like mammalian ER-phagy receptor FAM134B (212), Atg40 preferentially sits in regions of high membrane curvature, in which it can interact with Atg8 clusters present on the phagophore membrane to form multimeric Atg40 assemblies that deform the ER membrane. For receptors lacking reticulon-homology domains or transmembrane domains, clustering and activation of ER fragmentation may rely on the association with membrane proteins containing reticulon-homology domains or other motifs, as shown for example for SEC62, which forms heterodimeric complexes with FAM134C (204, 312).

In yeast, formation of ER-derived vesicles may involve the ER membrane protein Lnp1 (165), the lipid transfer protein Vps13 (313), and the multiple players linking the endocytic pits and the cortical ER sheets (314). These factors play still unclear roles in one or more events that eventually promote ER fragmentation and sequestration by autophagosomes. In this regard, the cytoskeleton and motor proteins may play a role in the scission of the ER subdomains from the main body. A model for yeast has been proposed in which, through the action of the End3-Pan1 and Arp2/3 complexes, which form a branched actin network at the endocytic pits (314), actin assembly promotes the invagination of endocytic pits (315) and their membrane contact site link with the rim of a cortical ER sheet, provided by the VAP proteins Scs2 or Scs22, the oxysterol-binding proteins Osh2 or Osh3, and the myosins Myo3 or Myo5 (316), serves as a tether to pull fragments of the cortical ER bearing Atg40 (314). Consistently, these events take place before the association of Atg40 with Atg11 and before the sequestration of the ER fragments within autophagosomes (314). This notion is also supported by earlier reports showing that blocking actin polymerization with latrunculin A prevents the colocalization of Atg40 with Atg11 (165) and inhibits ER-phagy (317).

Among the best candidate proteins to actively operate scission events are ATLs, which display a cytosolic dynamin-like GTPase domain (210, 211). ATLs are required for ER vesiculation (213), and their deletion, as shown for ATL2, inhibits FAM134B-regulated lysosomal degradation of ER sheets in cells exposed to nutrient deprivation (214). Nonetheless, it cannot be excluded that other cellular factors could mediate membrane scission, like for example dynamins, which have been shown to mediate mitochondria and peroxisome fission during mitophagy and pexophagy, respectively (318–320).

### 3.3. Triggering ER-Phagy Responses

ER-phagy operates constitutively, as shown by ER swelling upon deletion of determined ER-phagy receptors (200, 222). Importantly, a variety of signals elicit ER-phagy responses that enhance lysosomal ER turnover upon induction of ER-phagy receptor expression and/or activation. All in all, ER-phagy responses ensure the catabolic control of ER content, size, and functions and contribute with the anabolic unfolded protein responses to regulation of ER physiology in eukaryotic cells (5). Pleiotropic signals that trigger ER-phagy responses include nutrient restriction and pathological cellular stresses (sect. 3.3.1), which can be induced by pharmacological or chemical perturbation of the redox, protein *N*-glycosylation, or calcium homeostasis and may eventually result in cell death. They activate bulk autophagy and several types of organellophagy, including ER-phagy, in yeast, plant, and mammalian cells (84, 163–165, 185, 200, 208, 214, 228, 231, 236, 238, 248–250, 278, 290, 294, 301, 305, 317, 321–325). The ER-centric signals that trigger ER-phagy responses (sect. 3.3.2) originate from within the ER and include recovery from ER stress, accumulation of misfolded polypeptides, stalling of ribosomes, and pathogen invasion. They elicit lysosomal degradation of select ER portions, leaving unaffected cytoplasmic macromolecules and other organelles (45, 46, 186, 197, 198, 207, 209, 259, 260, 262, 282, 298, 326–330).

#### 3.3.1. Pleiotropic signals inducing ER-phagy in the context of bulk autophagy activation.

##### 3.3.1.1. nutrient restriction.

Metazoan cells and lower, nonphotosynthetic Eukarya get the energy needed for life from oxidation of complex organic molecules, including sugars, lipids, and proteins, harvested from the surrounding environment. Upon shortage of extracellular nutrients, cell survival relies on activation of autophagic programs in which part of the cytoplasmic content is degraded into basic metabolites inside lysosomes/vacuoles to generate an internal pool of nutrients (53, 103).

###### 3.3.1.1.1. Defining nutrient restriction.

“Nutrient restriction” is a vague term, as a multitude of metabolites are considered nutrients, and consequently this description does not specify what the cell or the organism is missing. Each metabolite has a different relevance, and its depletion leads to a specific cellular response, allowing the cell to adapt to and cope with its low levels. When several nutrients are simultaneously running out, then the cellular response becomes an intricated and coordinated sum of several cellular responses. Thus, the mode of induction, the activity, and the selectivity of autophagy vary depending on the type of nutrient restriction. Apparent discrepancies in the literature on autophagy regulation and/or progression in response to nutrient restriction, can be due to how the experiments were executed, that is, which metabolites were removed from the cell culture medium.

Historically, nutrient restriction has always been very well defined in yeast, already from the initial pioneering investigations in this organism, in which autophagy was investigated in response to nitrogen or carbon starvation (331, 332). Since then, most of the studies on autophagy mechanism and regulation in yeast have used nitrogen starvation, as this stress triggers autophagy almost immediately. Nonetheless, there have also been investigations in which autophagy was induced through carbon deprivation, and these have highlighted how autophagy induction differs depending on the cue (e.g., Refs. 333, 334). Interestingly, depletion of phosphate, zinc, or iron can also stimulate autophagy in yeast (335, 336), but only during phosphate starvation does autophagy appear to be selective in nature because requiring Atg11 (335).

Nutrient restriction in mammalian cells is really where this term becomes more heterogeneous in its significance. Under this terminology, researchers have studied autophagy by depriving cells of glucose, amino acids, or serum. Often experiments have also been carried out by depleting glucose and amino acids or amino acids and serum in combination or even just transferring cells in a buffer and thus removing all nutrients. In this plethora of conditions, it is imaginable that autophagy and all its subtypes are differently regulated and activated.

###### 3.3.1.1.2. Nutrient restriction and macro-ER-phagy induction.

As described in sect. 2.1.1.1, numerous signaling cascades regulating autophagy, including those sensing nutrient levels, modulate the activity of the Atg1/ULK kinase complex (74). A key regulator of the Atg1/ULK kinase complex activity is mTORC1, a kinase complex that orchestrates cell metabolism in response to a variety of upstream signals, including growth factors and nutrients such as amino acids (337). In the presence of these signals, mTORC1 represses macro-autophagy through direct phosphorylation of Atg1/ULK1 and Atg13/ATG13 (74, 75). Removal of amino acids, for example, leads to an inactivation of mTORC1 and concomitant dephosphorylation of its substrates, including Atg1/ULK1 and Atg13/ATG13 (74, 75). Removal of this posttranslational modification triggers Atg1/ULK kinase complex activation, initiating macro-autophagy (74, 75). The ULK kinase complex is also directly and positively regulated by the AMPK, which senses the cellular energy status and is activated when intracellular AMP increases, reflecting a decrease in availability of ATP (74, 75).

**FIGURE 11.**
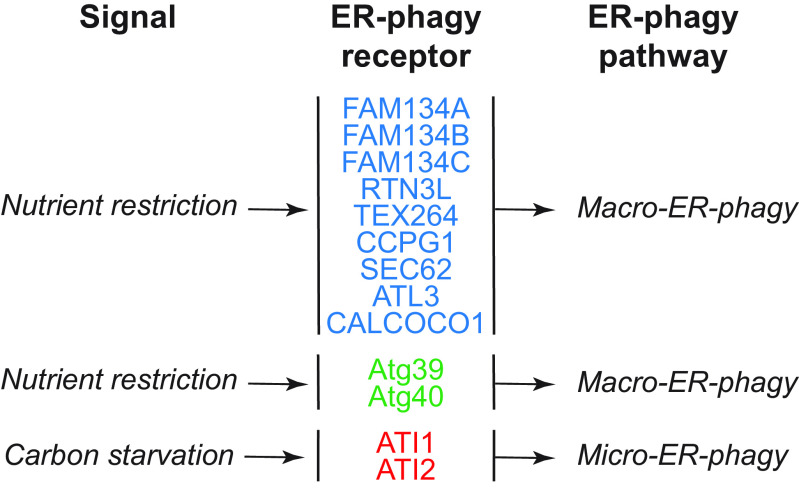
Documented pleiotropic cues activating ER-phagy responses: nutrient depletions. They generate an internal pool of nutrients required for cell/organism survival. Pleiotropic signals triggered by nutrient starvation induce macro-ER-phagy, which is characterized by the sequestration of ER fragments within autophagosomes carrying bulk cargoes. This type of macro-ER-phagy involves most of the known ER-phagy receptors. Nutrient restrictions can also elicit other ER-phagy pathways, as shown in plants during light-dark cycles that result in carbon starvation. Mammalian, yeast, and plant ER-phagy receptors are in blue, green, and red, respectively. See glossary for abbreviations.

Although bulk macro-autophagy is considered to be nonselective, pioneering studies in yeast on this process already revealed that determined molecules, like the Ape1 oligomers, are preferentially sequestered within autophagosomes also under nutrient starvation conditions, although the autophagosomal cargo is heterogeneous in composition (338). This is due to the fact that Ape1 oligomers are specifically recognized by the autophagy receptors Atg19 and Atg34, which allows a more effective sequestration within the forming autophagosomes in comparison to bulk substrates (339). This notion also applies to organelles, including the ER. A study in yeast was one of the first investigations to reveal that ER fragments are also targeted by autophagosomes under nitrogen starvation conditions or upon rapamycin treatment (317). Interestingly, the same study showed that carbon starvation enhanced ER turnover by bulk autophagy, highlighting that selective macro-autophagy programs can be differentially modulated depending on the conditions used to induce bulk macro-autophagy (317). These initial observations have provided the conceptual framework for subsequent works that, using the same conditions, have allowed identification of key factors and the mechanisms underlying macro-ER-phagy in yeast (84, 164, 165, 292, 313) and in plant cells (278, 294, 297, 305). The strategy of using nutrient starvation as an experimental setup to study macro-ER-phagy has been proven to be effective in mammalian cells as well (FIGURE 11). Most of the ER-phagy receptors identified so far, including FAM134B, RTN3L, CCPG1, and ATL3, have been initially characterized under conditions of amino acid depletion (248), pharmacological mTORC1 inhibition (248), or simultaneous serum and amino acid deprivation (163, 200, 213, 214, 228, 236).

These studies also showed the involvement of Atg8/LC3 lipidation and of the autophagosome biogenesis machineries in starvation-induced ER-phagy. Moreover, they have also shown that ER-phagy receptors identified in other contexts, e.g., SEC62, also participate in nutrient depletion-induced macro-ER-phagy (FIGURE 11) (231). Notably, ER-phagy clears a small fraction of the cellular ER, which is the largest organelle in nucleated cells. This has been quantified in consumption of <10% of ER reporter proteins in cells subjected to 10 h of amino acid starvation (231, 248).

##### 3.3.1.2. pathological cellular stresses also impacting on er functions.

###### 3.3.1.2.1. Defining the cellular stresses.

**FIGURE 12.**
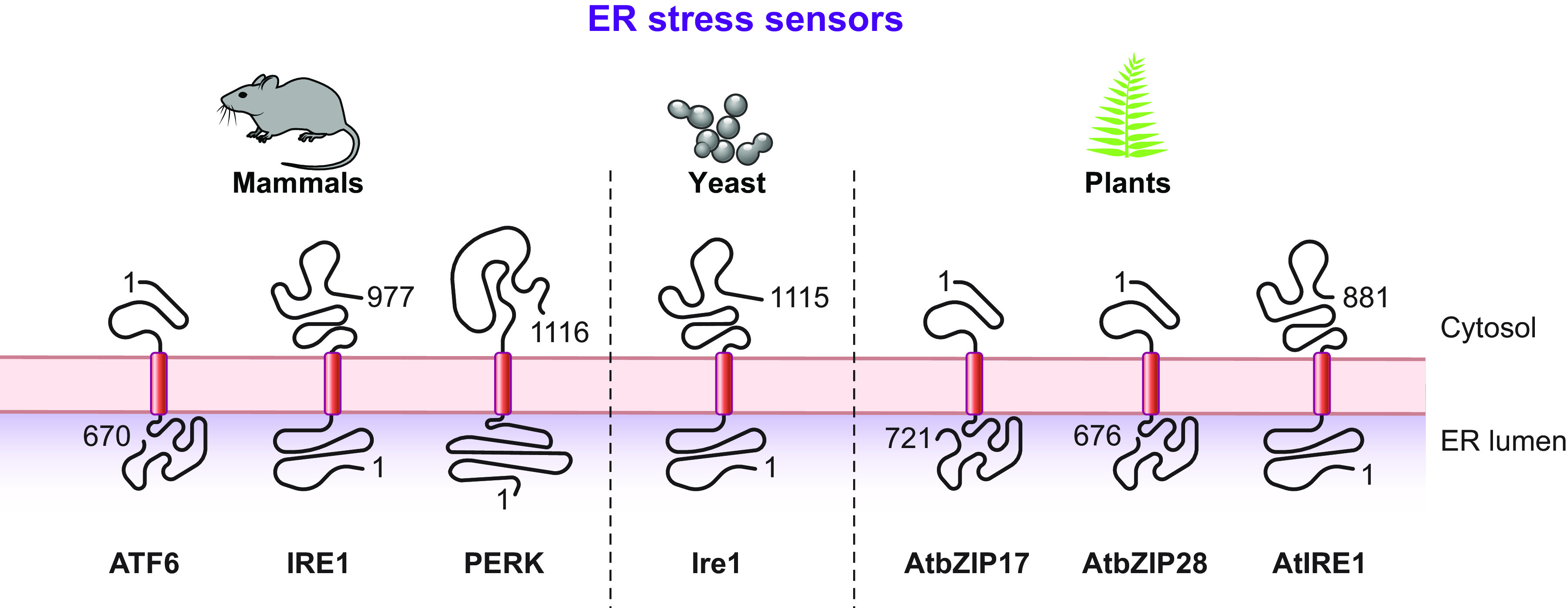
ER stress sensors. AtbZIP17 and AtbZIP28 are the plant homologs of mammalian ATF6. See glossary for abbreviations.

Perturbations of cellular homeostasis activate the posttranslational modification of a set of ER membrane proteins collectively called ER stress sensors (FIGURE 12): IRE1, ATF6, and PERK in Metazoa; AtbZIP17 and AtbZIP28, the ATF6 homologs, and the IRE1 ortholog in plants; and Ire1 in yeast. The posttranslational modifications are oligomerization and phosphorylation in the case of the IRE1 proteins and PERK and proteolytic processing for the ATF6 proteins.

Posttranslational modifications are elicited by physiological signals such as cell differentiation, e.g., differentiation from quiescent B cells to antibody-secreting plasma cells (340), generation of highly secretory organs like the liver (340), and dark to light cycles in plants (341). They are also triggered by pathological signals such as pathogen infections (342), challenges with chemical compounds that perturb protein *N*-glycosylation, calcium or redox homeostasis (343), and accumulation of misfolded proteins (344–346). Activation of the ER stress sensors triggers downstream signal-specific transcriptional and translational programs, which differ depending on the cues (344–346). These unfolded protein responses eventually increase the ER size, the levels of the ER-resident proteins and enzymes, and the overall ER activities. If ER homeostatic perturbations are persistent, they may eventually trigger adaptation; if they are harmful, they initiate proapoptotic programs (347). The vast knowledge available on unfolded protein responses has been exhaustively reviewed elsewhere (2–4).

###### 3.3.1.2.2. Stress-induced macro- and micro-ER-phagy.

**FIGURE 13.**
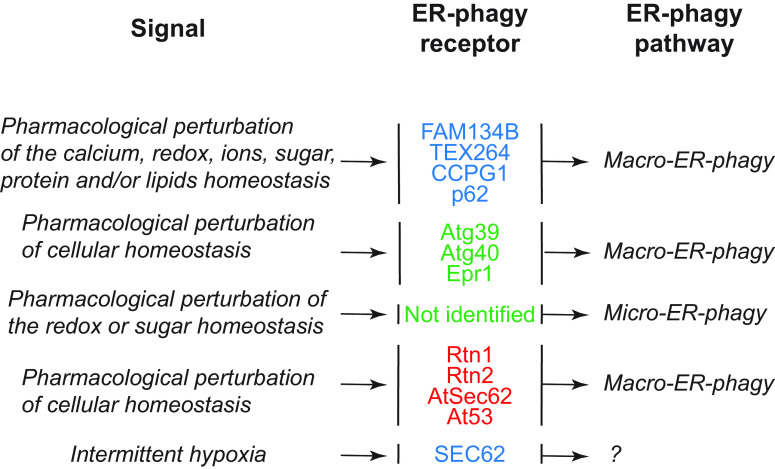
Documented pleiotropic cues activating ER-phagy responses: cellular stresses. They control ER expansion and/or eliminate ER portions containing damaged, aged, and/or toxic material. These pleiotropic signals are triggered by perturbation of calcium, redox, ions, sugar, protein and/or lipid homeostasis. In addition to inducing a variety of cell responses, this type of pleiotropic signals activates macro-ER-phagy, which is characterized by sequestration of ER fragments within autophagosome carrying bulk cargoes via unfolded protein responses. In yeast, direct capture of ER whorls by the vacuole through micro-ER-phagy has been reported. Mammalian, yeast, and plant ER-phagy receptors are in blue, green and red, respectively. See glossary for abbreviations.

Unfolded protein responses and ER-phagy responses ensure the anabolic and the catabolic regulation of the ER size and functions. Not unexpectedly, their activities and regulation are closely interconnected, as supported by strong experimental evidence. Pharmacological and chemical perturbation of mammalian, yeast, and plant cell homeostasis may induce unfolded protein responses. These may in turn potently enhance lysosomal clearance of ER portions to prevent excessive ER expansion and thus protect cells from apoptosis (FIGURE 13) (260, 262, 293, 301, 323, 325, 348). Enhanced ER-phagy under these experimental conditions appears in some cases to be a consequence of the induced expression of individual ER-phagy receptors such as mammalian FAM134B, TEX264 (218), and CCPG1 (163), yeast Atg39, Atg40, and Epr1 (290, 293), and plant cell Rtn1 and Rtn2 (238).

The close interconnection between unfolded protein responses and ER-phagy responses is also supported by the fact that defective activation or progression of starvation-induced ER-phagy results in substantial ER stress (231) and that ER-phagy responses, namely in this case recov-ER-phagy (for ER-phagy during recovery from ER stress), may also be activated at the end of a UPR to resume physiological ER size and functions (45, 46, 186). All in all, the plasticity of the ER that warrants rapid adaptation to changing homeostatic needs is ensured by the concerted activity of the catabolic ER-phagy responses and the anabolic unfolded protein responses, whose mechanistic details remain to be investigated. Mechanistically, ER stress-induced ER-phagy may proceed via macro-ER-phagy, i.e., via sequestration of ER fragments by autophagosomes or, at least in yeast, via micro-ER-phagy, upon formation of ER whorls that are directly engulfed by the vacuole with intervention of the ESCRT machinery (64, 184, 185).

#### 3.3.2. ER-centric signals inducing selective ER-phagy.

##### 3.3.2.1. recovery from er stresses.

**FIGURE 14.**
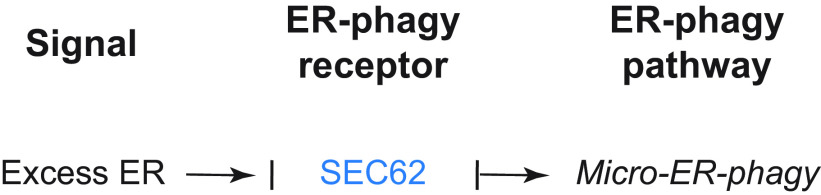
Documented ER-centric cues activating ER-phagy responses: recovery from ER stresses (recover-ER-phagy). It removes excess ER. The mammalian ER-phagy receptor is in blue. See glossary for abbreviations.

A role for ER-phagy during cell recovery from pharmacologically induced ER stresses was postulated by pioneering works reporting that upon the interruption of administration of the antiepileptic drug phenobarbital to albino rats, which induces expression of drug-detoxifying enzymes and swelling of the smooth ER in the animal’s hepatocytes (349), excess ER is selectively delivered to lysosomes to recover physiological size and activity of the biosynthetic compartment (42). Since then, the selective lysosomal delivery of portions of the ER, and not of other organelles, during recovery from ER stresses has been reproduced in different cultured cell lines and animal models (e.g., Refs. 45, 46, 186, 350, 351). Recent studies confirmed that this type of ER-phagy, named recov-ER-phagy, is induced upon interruption of pharmacological treatments causing ER stresses through perturbations of calcium or redox homeostasis (FIGURE 14) (186). Removal of excess ER during recovery from ER stress relies on the fragmentation of ER portions that contain stress-induced ER-resident chaperones as well as enzymes of the protein disulfide isomerase superfamily but lack ER-associated degradation (ERAD) factors. Hence, the exquisite selectivity of this catabolic program is testified to by both the lack of delivery of other organelles to the degradative districts and the preservation of ER subdomains that ensure selection of misfolded polypeptides for ERAD (186). Note that the catabolic regulation of ERAD factors relies on ERAD tuning pathways described elsewhere (352–355).

Recov-ER-phagy is regulated by the ER-phagy receptor SEC62 (FIGURE 14) (sect. 3.2.1.1.2), which has a well-established role in the ER membrane. That is, it regulates the posttranslational translocation of newly synthesized secretory proteins in the ER by participating in a heterodimeric complex with SEC63 that associates with the SEC61 protein translocation channel (356). The findings that silencing of SEC63 or the induction of SEC62 expression recapitulates recov-ER-phagy at steady state imply that the SEC62-SEC63 complexes involved in protein translocation may be disassembled during recovery from ER stresses and the orphan SEC62 proteins expose their LIR motifs to engage the cytosolic machinery mediating ER-phagy (45, 46, 186). Morphologically, recov-ER-phagy is a type II micro-autophagy (FIGURE 14), in which ER fragments are directly captured by LAMP1/RAB7-positive endolysosomal compartments, as shown by immuno-EM micrographs (45, 46, 186). Supporting the morphological analyses, genetic and pharmacological evidence revealed that recov-ER-phagy relies on *ATG* genes that control LC3 protein lipidation, whereas other core *ATG* genes, namely those involved in autophagosome biogenesis, are dispensable (186). This implies that LC3 proteins are lipidated onto single-membrane organelles (i.e., the ER itself, vesicles that are released from the ER, or the target degradative compartment), and it is conceptually consistent with an increasing number of reported situations in which single-membrane organelles are the site of LC3 lipidation. These situations include those resulting upon STING activation in response to viral or bacterial attack (263), LC3-associated phagocytosis induced by bacterial and fungal engulfment (357–359), engulfment of apoptotic cells (i.e., entotic cell clearance) (360), engagement of Toll-like receptors on macrophages (361), cell exposure to bacterial toxins (e.g., the vacuolating toxin of *Helicobacter pylori*), and topical application on skin of anesthetic (i.e., lidocaine) or subministration of the FDA-approved amiodarone to treat cardiac dysrhythmias, which induce vacuolation of single-membrane acidic structures (362–367), which are LC3 positive (363), and many others. In recov-ER-phagy, components of the ESCRT-III complex, including the AAA^+^ ATPase VPS4A and CHMP4B, ensure micro-autophagic capture of ER-derived vesicles by the endolysosomal compartments (45, 46, 186). All in all, SEC62-driven recover-ER-phagy is clearly distinct from the ER-phagy responses elicited by pleiotropic signals, in which SEC62 also intervenes (231) (sect. 3.3.1). In fact, nutrient deprivation or pathological ER stresses activate most, if not all, ER-phagy receptors, including FAM134 proteins, SEC62, TEX264, ATL3, CALCOCO1, RTN3L, and CCPG1, to initiate macro-ER-phagy (163, 200, 208, 214, 228, 231, 236, 248–250). These findings highlight the concept that the mechanisms ensuring ER-phagy are determined by the tissue and cellular contexts in which the ER portions to be cleared are located and the cues that elicit the “eat-me” program.

##### 3.3.2.2. accumulation of misfolded proteins in the er.

**FIGURE 15.**
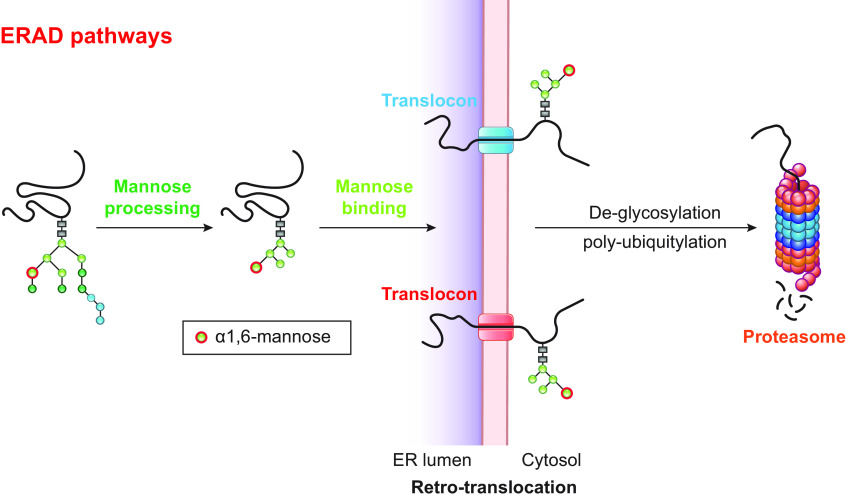
ER-associated degradation and N-glycan processing. Slow mannose removal from N-glycans on terminally misfolded proteins by the ER-α-mannosidase I and EDEM glycosidases (mannose processing) generates N-glycans that display a terminal α1,6-bonded mannose residue (red circle). This is recognized by lectins such as OS-9 and ERLECTIN1 (mannose binding). These 2 lectins shuttle extensively demannosylated, terminally misfolded proteins to client-specific supramolecular complexes including membrane-embedded E3 Ub ligases (translocon). Retrotranslocation into the cytoplasm is followed by deglycosylation, polyubiquitylation, and proteasomal degradation. See glossary for abbreviations.

Misfolded proteins must rapidly be removed from the ER to avoid cytotoxic effects. Most of them are either dislocated across or extracted from the ER membrane, polyubiquitylated, and finally degraded by cytosolic proteasomes through catabolic processes collectively defined as ER-associated degradation (ERAD) (FIGURE 15). Misfolded polypeptides that fail to enter the ERAD systems are segregated in dedicated ER subdomains on the intervention of ER-resident chaperones, which in turn engage ER-phagy receptors (section 3.2.1). This activates ER-phagy responses that promote vesiculation of these ER portions and their subsequent lysosomal degradation. These processes have been referred to as ER-to-lysosome-associated degradation (or ERLAD) (44, 48, 368–372).

###### 3.3.2.2.1. Proteasomal degradation of misfolded proteins from the ER.

The ER is a major site of protein synthesis and folding. About 40% of the gene products enter during, or just after, their synthesis in the ER of nucleated cells. They complete their folding programs under the assistance of ER-resident molecular chaperones and folding factors and are eventually transported to the appropriate intra- or extracellular site of activity (373). A dedicated quality control machinery inspects newly synthesized proteins to retain and selects the defective ones for degradation, via dedicated ERAD pathways (368, 369). The finding that brefeldin A, a macrolide antibiotic that inhibits protein trafficking from the ER to the Golgi, does not hamper clearance of misfolded polypeptides initially suggested that ERAD occurs within the ER lumen (374).

**FIGURE 16.**
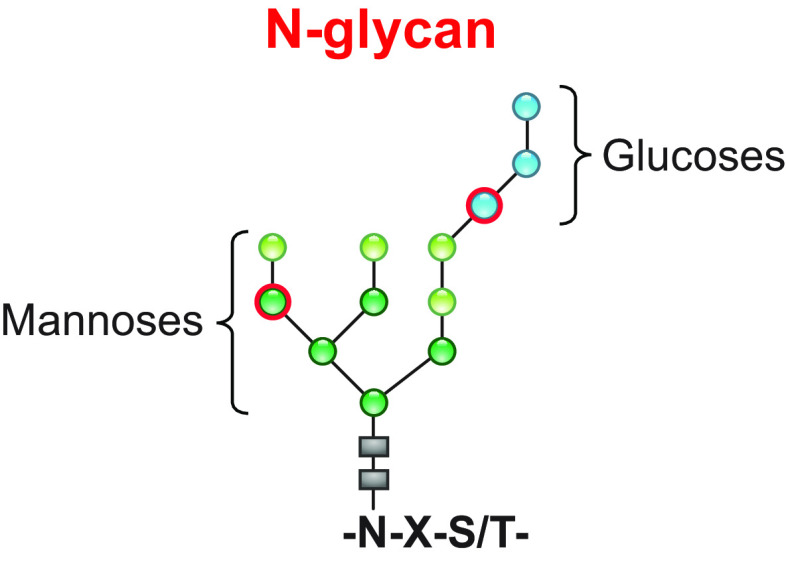
Structure of the N-linked glycans. These oligosaccharides are preassembled on a dolichol lipid moiety and conjugated en bloc to an Asn residue in the consensus sequence Asn-X-Ser/Thr (N-X-S/T) of newly synthesized proteins, in which X can be any amino acid except a proline. N-glycans can be found on ER luminal proteins and in the luminal domains of integral ER membrane polypeptides. Inside the red-outlined circles, the green mannose residue elicits ERAD and the blue glucose residue elicits ERLAD.

The following years were decisive to uncovering that ERAD requires the retrotranslocation of misfolded polypeptides across the ER membrane (for luminal proteins) or their extraction from the ER membrane (for integral membrane proteins) but also ubiquitylating enzymes and the cytosolic 26S proteasomes (375–382). Since then, the mechanisms of selection of terminally misfolded polypeptides and their dislocation across the ER membrane, polyubiquitylation, deglycosylation, and proteasomal degradation have been dissected in molecular details in both yeast and mammalian cells. Moreover, it has been shown that the ERAD consists of numerous pathways that rely on client-specific engagement of translocation machineries built around several membrane-embedded E3 ubiquitin ligases and all converging to the cytosolic proteasomes (FIGURE 15) (5, 368, 369, 383–386). In the case of glycoproteins, which represent the vast majority of the gene products synthesized in the ER, the persistent retention in the ER as a consequence of failed folding attempts leads to an extensive removal of mannose residues from their N-linked oligosaccharides (FIGURES 15 and 16), which is performed by members of the glycosyl hydrolase family 47 that includes ER-α-mannosidase I and ER degradation-enhancing α-mannosidase-like (EDEM) proteins (FIGURE 15, Mannose processing) (387, 388). Mannose trimming generates ligands for the mannose-binding proteins osteosarcoma amplified 9 (OS-9) and Erlectin1 (FIGURE 15, Mannose binding) (369, 389–392), which convey the misfolded polypeptides to multimeric protein complexes, the translocation (or retrotranslocation) machineries.

These are built around transmembrane E3 Ub ligases and probably work synergistically with members of the rhomboid protein family, including Derlins and intramembrane serine proteases (FIGURE 15, Retro-translocation) (387, 393, 394). The client-specific pathway engaged to ensure proteasomal clearance depends on the presence of protein-bound oligosaccharides (389, 390, 395–397), the topology of the misfolded protein (i.e., membrane bound vs. soluble) (392, 398, 399), the position of the folding defect (i.e., in the luminal, intramembrane, or cytosolic portion of the polypeptide chain) (400, 401), the content in disulfide bonds (399, 402–407), and/or the peptidyl-prolyl bonds in the *cis* conformation (408). Several reviews have covered all these issues comprehensively (368, 369, 383, 385, 409).

###### 3.3.2.2.2. Lysosomal degradation of ER portions containing misfolded proteins.

**FIGURE 17.**
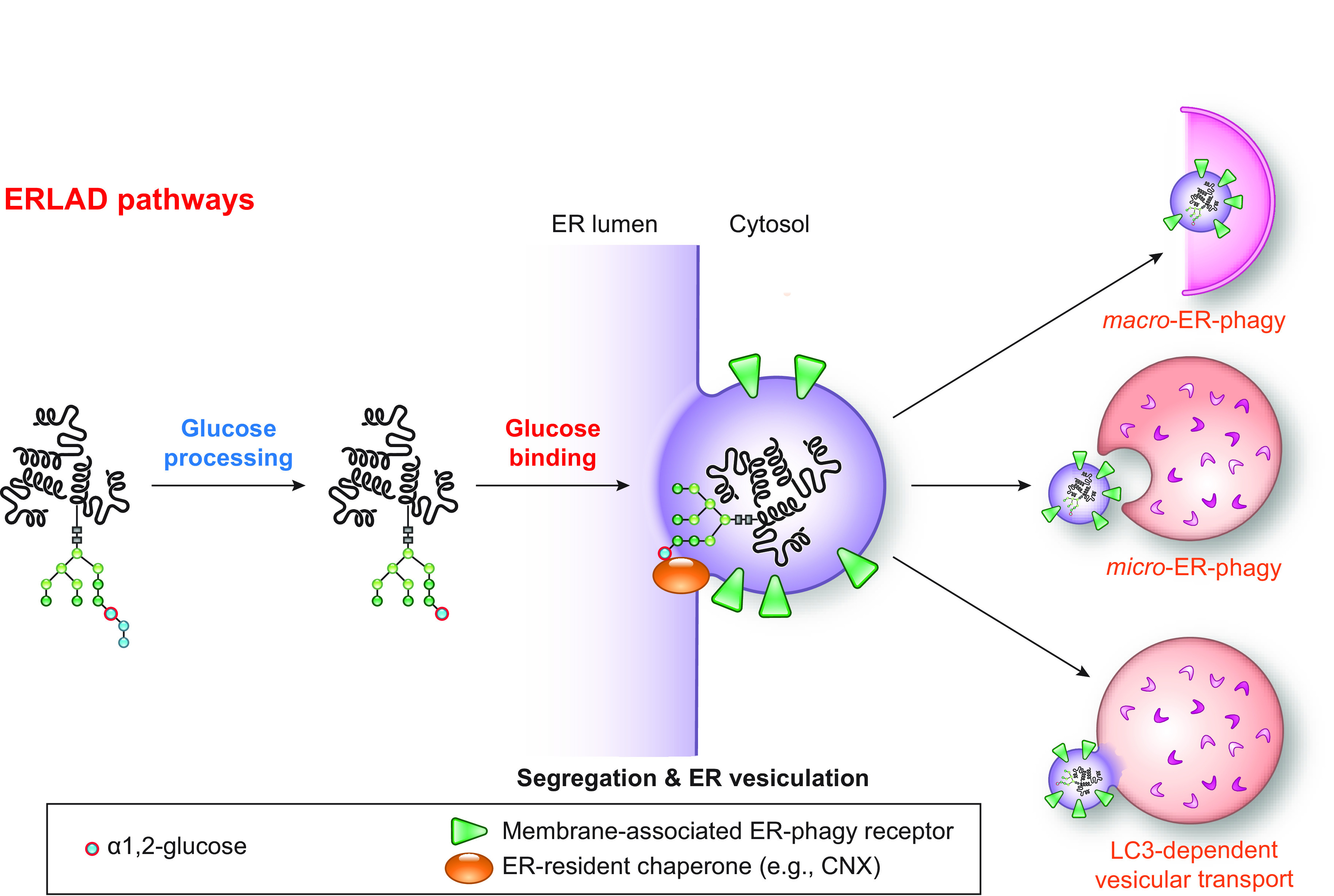
ER-to-lysosome-associated degradation (ERLAD) and N-glycan processing. Cycles of glucose removal by ER-resident α-glucosidases and glucose re-addition by UGGT1 (glucose processing) retain terminally misfolded proteins associated with CNX. The glucose residue ensuring CNX binding is shown as a red circle. CNX acts as a cargo receptor that, through interaction with specific ER-phagy receptors, elicits ERLAD. ER-to-lysosome-associated degradation is characterized by cargo segregation in determined regions of the ER, vesiculation/fragmentation of this discrete ER regions, and their turnover by macro-ER-phagy, micro-ER-phagy, or LC3-dependent vesicular transport to lysosomes/vacuoles. See glossary for abbreviations.

Misfolded polypeptides may fail to enter the ERAD pathways. The large size and the propensity to form aggregates or polymeric structures can impede the polypeptide’s transport across the ER membrane, which is necessary to become exposed to polyubiquitylation and proteasomal degradation. Persistent association with ER-resident chaperones may also retain defective gene products in the ER lumen and impede retrotranslocation into the cytosol. The lack of association with chaperones that convey misfolded proteins to retrotranslocation sites at the ER membrane is yet another aspect that may contribute to determined misfolded polypeptides not being selected for ERAD (368). Removal of these faulty gene products relies on their segregation in ER subdomains that are eventually cleared from cells by ER-to-lysosome-associated degradation (ERLAD) (FIGURE 17).

**FIGURE 18.**
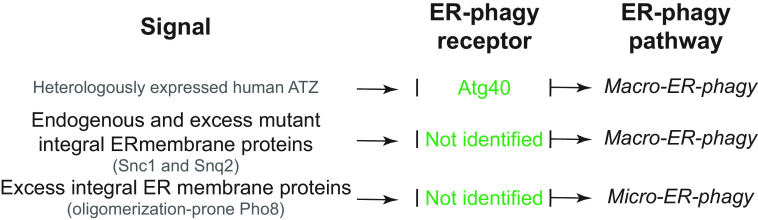
Documented ER-centric cues activating ER-phagy responses: accumulation of misfolded proteins in the yeast ER. These ER-to-vacuole-associated degradation pathways remove aberrant gene products. Select examples are indicated in gray. Yeast ER-phagy receptors are in green. See glossary for abbreviations.

A rapidly expanding number of misfolded yeast and mammalian proteins accumulating in the ER have been shown to rely for clearance on ER-to-lysosome-associated degradation. In yeast, misfolded protein segregation in ER subdomains eventually cleared in vacuoles include mammalian folding-defective proteins used as model substrates such as ATZ, the aggregated cystic fibrosis transmembrane conductance regulator (CFTR), and the bovine pancreatic trypsin inhibitor (292, 410–413), as well as endogenous, overexpressed, and artificially designed membrane proteins (FIGURE 18). These include oligomerization-prone proteins, such as Pho8, that induce formation of ER whorls that are eventually cleared via micro-ER-phagy, proteins carrying mutations in their intramembrane domains such as Snc1, or multispanning proteins like Snq2 that are removed from cells by ER-to-vacuole associated degradation via macro-ER-phagy (FIGURE 18) (185, 414–425).

**FIGURE 19.**
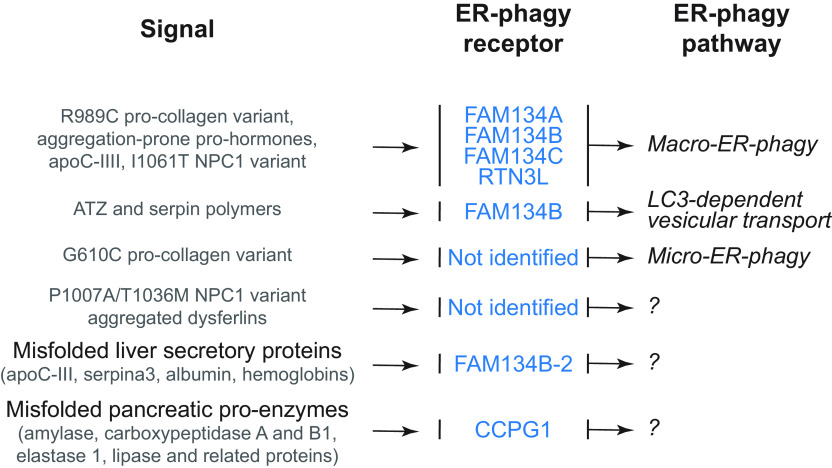
Documented ER-centric cues activating ER-phagy responses: accumulation of misfolded proteins in the mammalian ER. These ER-to-lysosome-associated degradation pathways remove aberrant gene products. The engaged ER receptors and ER-phagy pathway depend on the client, cell type, and/or tissue. Select examples are indicated in gray. Mammalian ER-phagy receptors are in blue. See glossary for abbreviations.

In mammalian cells, examples are serpin’s polymers (197, 198, 292, 426–435), β-subunits of thyrotrophic hormone (193), procollagen variants (198, 199, 207, 216, 326, 330, 436), mutant dysferlins (437), mutant forms of the Niemann–Pick type C protein (209), mutant conformers of gonadotropin-releasing hormone receptor (438), mutant forms of the cystic fibrosis transmembrane conductance regulator (439), various liver and pancreas secretory enzymes and proenzymes (163, 208), aggregation-prone prohormones (pro-insulin, pro-opiomelanocortin, pro-arginine-vasopressin) (440, 441), thyroglobulin (442), BEST1 (443), prion (444–446), progressive ankylosis protein (447), defective mutant fibrinogen (448), and apolipoprotein C-III (208) (FIGURE 19). In most cases, delivery of these misfolded proteins from the ER to the endolysosomal degradative compartments bypasses the Golgi, which is the traffic route used to transport lysosomal enzymes from the ER to their destination. In contrast to ERAD, the mechanistic dissection of the pathways ensuring lysosomal removal of portions of the ER containing misfolded proteins is in its infancy and the scarce published information refers to a handful of client proteins. Available data reveal that the misfolded polypeptides are segregated from folding intermediates and concentrated into ER subdomains (FIGURE 17, Segregation), which are eventually separated from the bulk ER (FIGURE 17, ER vesiculation) and cleared from cells by a collection of ER-to-lysosome-associated degradation pathways (or ER-to-vacuole-associated pathways in yeast and plants) (5). These misfolded protein-containing ER-derived vesicles/fragments can be captured by autophagosomes, be engulfed by endolysosomal compartments, or directly fuse with the endolysosomal compartments (FIGURE 17) (reviewed in Refs. 5, 47, 48, 368–371, 449). In some specific cases, like for prion proteins in mammalian cells and selected misfolded membrane proteins in yeast, the delivery to the lysosomal/vacuolar district for destruction follows the conventional secretory route (419, 422–425, 444, 446, 450–452).

####### 3.3.2.2.2.1. er-to-vacuole-associated degradation in yeast.

In yeast, aberrant gene products that fail to engage the ERAD machinery can be selectively delivered to the vacuole (411, 420). This may proceed via macro-ER-phagy as shown by the involvement of gene products required for Atg8 lipidation and for autophagosome biogenesis in degradation of excess membrane proteins (412, 416, 418) and/or by micro-ER-phagy, in which *ATG* gene products and autophagosome intervention are dispensable and the vacuole directly engulfs ER portions containing misfolded proteins with intervention of the ESCRT machinery (FIGURE 18) (64, 184, 185). In contrast to starvation-induced macro-ER-phagy, which requires the ER-phagy receptors Atg39 and Atg40, macro-ER-phagy ensuring ER-to-vacuole-associated degradation can and cannot involve ER-phagy receptors. More precisely, ER-to-vacuole-associated degradation of ER portions containing polymers formed by the Z variant of α1-antitrypsin (ATZ, a human protein also used to characterize yeast ER-to-vacuole-associated degradation pathways) involves the ER-phagy receptors (292). In contrast, turnover of ER portions containing excess integral ER membrane proteins does not involve the ER-phagy receptors identified so far and may proceed via macro-ER-phagy (415) or via micro-ER-phagy when the membrane-bound proteins oligomerize and induce formation of ER whorls (185) (FIGURE 18).

In the specific case of the mammalian protein ATZ expressed in yeast, clearance from the ER requires segregation into specialized ER exit sites, called ER-phagy sites, that are characterized by the presence of the unconventional COPII vesicle adaptor complex Lst1-Sec23 and Atg40 (292). This noncanonical coat on ER exit sites facilitates the vesiculation and the sequestration of ER fragments by the autophagosomes. The process is conserved in mammalian cells, since SEC24C, the paralog of Lst1, also participates in macro-ER-phagy (292). Altogether, these findings show that client-specific ER-to-vacuole-associated degradation pathways contribute to removal of misfolded proteins from the yeast ER.

####### 3.3.2.2.2.2. er-to-lysosome-associated degradation in mammalian cells.

The involvement of lysosomes in the clearance of misfolded proteins accumulated in the mammalian ER has long been established (43, 193, 453). These ER-to-lysosome-associated degradation pathways (FIGURES 17 and 19) have been studied extensively recently, and they concur with the ERAD pathways (FIGURE 15) in the maintenance of ER proteostasis (5, 47, 48, 368–371, 449). The cooperation of ER-to-lysosome-associated degradation and ERAD in the removal of defective gene products from the ER is highlighted by the cases of mutant forms of serpins and dysferlins, whose aggregates are degraded by ER-to-lysosome-associated degradation while monomers are turned over by ERAD (197, 426–434, 437).

Another example is the pathogenic variants of the multipass transmembrane protein Niemann–Pick type C (NPC1), which is partially degraded by FAM134B-driven ER-to-lysosome-associated degradation and partially by membrane-associated ring-CH-type finger 6 (MARCHF6)-driven ERAD (209).

####### 3.3.2.2.2.3. atg proteins in er-to-lysosome-associated degradation.

The findings that BECN1, ATG5, ATG7, or other ATG proteins participate in clearance of large and/or aggregated misfolded polypeptides generated in the ER of cultured cells (e.g., Refs. 426, 428, 436–438, 454) added genetic support to the pioneering works cited above (43, 193, 453) in which the intervention of lysosomes in the catabolic turnover of ER components was highlighted. These pioneering findings originated two models. In the first model, misfolded polypeptides are transported across the ER membrane into the cytoplasm, where they are sequestered by forming autophagosomes, to be eliminated through macro-autophagy (5, 433, 455). In the second model, misfolded proteins are segregated into ER subdomains, and these latter are delivered to the lysosomal district for clearance (5, 433, 455). Assessment of an expanding list of ER-to-lysosome-associated degradation clients shows that the second model applies (FIGURE 17). The involvement of *ATG* genes regulating LC3 lipidation has been confirmed for the lysosomal or vacuolar degradation of a variety of model misfolded polypeptides. In contrast, core ATG proteins mediating autophagosome biogenesis are dispensable for some clients. In these latter situations, ER-derived fragments containing the misfolded polypeptides and/or protein aggregates are either engulfed by endolysosomal compartments or directly fuse with them (FIGURE 17) (48, 368–371, 449).

####### 3.3.2.2.2.4. er-phagy receptors in er-to-lysosome-associated degradation.

The involvement of ER-phagy receptors in ER-to-lysosome-associated degradation (FIGURE 17) is supported by various findings. First, gene editing that inactivates FAM134B, FAM134A, and/or FAM134C impairs disposal of ATZ polymers (197, 198), misfolded pro-collagen (198, 199, 207), apolipoprotein C-III (208), and NPC1 variants (209) in cultured cells (FIGURE 19). Second, *CCPG1* hypomorphism in mice results in accumulation of insoluble acinar secretory enzymes (FIGURE 19) (163). Third, fasting mice lacking the liver-specific ER-phagy receptor FAM134B-2 display an intraluminal accumulation of >40 aberrant secretory proteins in the hepatocytes (FIGURE 19) (208). The case of RTN3L merits a note. The concomitant silencing of all the RTN3 isoforms results in the accumulation of ER-to-lysosome-associated degradation clients, such as the protein condensates and complexes formed in the ER lumen by proinsulin Akita, the C28F mutant of proopiomelanocortin, and the G57S variant of the neuropeptide pro-arginine-vasopressin (440, 456, 457). The finding that the ER-to-lysosome-associated degradation defect caused by the depletion of RTN3 proteins is rescued upon backtransfection of either the short form of RTN3 or RTN4, both of which lack LIR motifs, warrants further investigation (440). In this RTN3-driven ER-to-lysosome-associated degradation, the transmembrane ER protein PGRMC1, which in contrast to RTN3 proteins has a luminal domain, recruits the clients to be segregated in ER subdomains that are eventually delivered to the degradative compartments for clearance (307). An alternative model suggests a role of RTN3L as an ER-phagy receptor in these catabolic pathways, which rely on the segregation of the ER-to-lysosome-associated degradation clients in ER-phagy sites also displaying the COPII coat subunit SEC24C (457). Notably, SEC24C shows a specific interaction with RTN3L (236).

It starts to emerge that, like ERAD, in which the client’s features eventually determine the translocation machinery for the delivery into the cytoplasm and proteasomal degradation (FIGURE 15) (sect. 3.3.2.2.1) (369, 458, 459), client-specific and possibly redundant pathways operate in ER-to-lysosome (or ER-to-vacuole)-associated degradation as well (FIGURES 17 and 19) (5). As stated above, different misfolded polypeptides engage different ER-phagy receptors (FIGURE 19). Moreover, different mutants of the same protein are turned over by different ER-to-lysosome-associated degradation mechanisms. For example, the ER-to-lysosome-associated degradation of the I1061T mutant of NPC1 is driven by FAM134B, whereas that of the P1007A/T1036M NPC1 variant is not (FIGURE 19) (209). Even more astonishingly, engagement of the same ER-phagy receptor by different clients can trigger distinct ER-to-lysosome-associated degradation pathways. For example, the engagement of the FAM134-CNX complexes by aberrant ATZ polymers elicits formation of LC3-positive ER-derived vesicles that directly fuse with endolysosomal compartments (FIGURE 19, LC3-dependent vesicular transport) (197, 198). In contrast, the engagement of the FAM134-CNX complexes by mutant forms of pro-collagen triggers the formation of ER-derived fragments that are captured by nascent autophagosomes (FIGURE 19, Macro-ER-phagy) (198, 207). Notably, autophagosomes are dispensable for the clearance of ER portions containing other forms of misfolded pro-collagen, e.g., the G610C mutant, which is segregated in osteoblasts’ and osteoclasts’ ER exit sites and turned over by micro-ER-phagy (FIGURE 19, Micro-ER-phagy) (326, 329, 330).

####### 3.3.2.2.2.5. n-glycan processing in erad and in er-to-lysosome-associated degradation.

The ER membrane contains ER stress sensors and ER-phagy receptors that activate unfolded protein responses and ER-phagy responses, respectively. The major difference between these two classes of signal transducers is that ER stress sensors have large intraluminal domains that bind misfolded polypeptides or the free molecular chaperone BiP and directly report on changes in ER homeostasis (4) (FIGURE 12). In contrast, intraluminal domains are virtually absent in most ER-phagy receptors (FIGURES 8–10). Hence, the initiation of ER-to-lysosome-associated degradation programs relies on adaptor proteins that recognize the misfolded proteins in the ER lumen and directly or indirectly associate with select ER-phagy receptors. This promotes segregation of the misfolded polypeptides in subdomains of the ER that eventually vesiculate and are delivered to the endolysosomal compartments via macro-ER-phagy, micro-ER-phagy, and/or LC3-dependent vesicular transport (FIGURE 17). Most notably, CNX is a major interactor of mammalian FAM134 protein family members (197, 200), and its role in ER-to-lysosome-associated degradation of both ATZ and misfolded pro-collagen has recently been established in molecular details (197, 198, 207). Briefly, most proteins entering the ER are covalently modified with mannose- and glucose-containing oligosaccharides conjugated to asparagine residues (FIGURE 16). This proceeds both cotranslationally and posttranslationally and is defined as protein’s N-glycosylation, where N stands for an asparagine residue (387) (FIGURE 16). Slow mannose removal from N-linked oligosaccharides selects terminally misfolded proteins for proteasomal degradation (FIGURE 15) (387, 388). As for ER-to-lysosome-associated degradation, it has recently been established that client selection relies on cycles of glucose removal by ER-resident α-glucosidases and glucose readdition by the UDP-glucose:glycoprotein glucosyltransferase I (UGGT1), resulting in persistent association of terminally misfolded polypeptides with the pool of CNX associated with FAM134B (FIGURE 17) (197, 198). ER-to-lysosome-associated degradation elicited through this mechanism proceeds via either LC3-dependent vesicular transport, in the case of ATZ polymers (197, 198), or macro-ER-phagy, in the case of mutant forms of pro-collagen (198, 207). Removal of N-glycans or genetic or pharmacological inactivation of the ER-resident α-glucosidases, UGGT1 and CNX, substantially abolishes selection of misfolded polypeptides for ER-to-lysosome-associated degradation (FIGURE 17) (198).

Intriguingly, for multiglycosylated polypeptides like ATZ, only one of the oligosaccharides, at amino acid position 83, signals for ER-to-lysosome-associated degradation. This finding confirms the functional prevalence of one oligosaccharide in the context of multiglycosylated proteins, something that has also been observed in N-glycan-mediated protein folding [e.g., only the oligosaccharide at position 81 is absolutely required for maturation of the hepta-glycosylated influenza virus hemagglutinin (460, 461)] and N-glycan-regulated ERAD [e.g., only one of their N-glycans is required for the turnover of mutant CPY* and PrA* (462)]. Since other mammalian and plant ER-phagy receptors form functional complexes with ER-resident lectin chaperones (sect. 3.2.4) (238, 248, 306, 463), N-glycosylation and N-glycan processing may play a relevant role in selection of misfolded polypeptides for segregation in ER subdomains destined to ER-to-vacuole or ER-to-lysosome-associated degradation.

##### 3.3.2.3. stalling of ribosomes.

**FIGURE 20.**
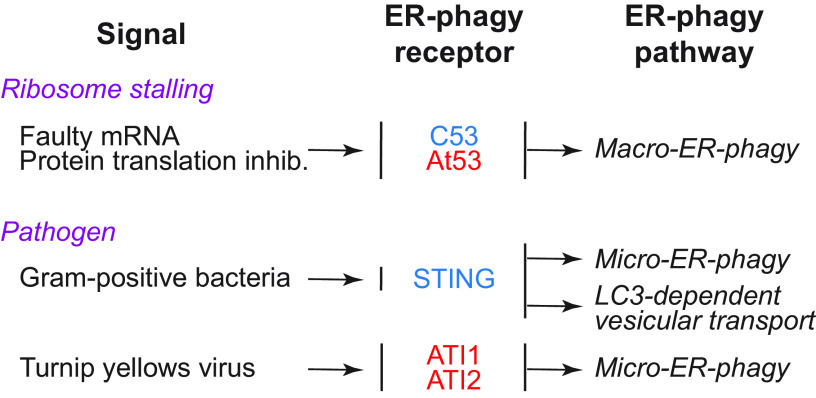
Documented ER-centric cues activating ER-phagy responses: ribosome stalling or pathogen invasion. They are important for the turnover of aberrant transcript, microbial gene products, or antimicrobial gene products (e.g., the Ago1 protein in *A. thaliana* cells infected with the Turnip yellows virus). Mammalian and plant ER-phagy receptors are in blue and red, respectively. See glossary for abbreviations.

Newly synthesized proteins are posttranslationally or cotranslationally translocated into the ER via the translocon (227). In both cases, the polypeptide can remain trapped in the translocon. Unclogging of the translocon from a protein being posttranslationally translocated requires the ER-associated zinc metalloprotease zinc metalloproteinase STE24 (ZMPSTE24), which proteolytically removes the obstructing polypeptide (368, 464). However, translocons can also be blocked during cotranslational protein import, when the ribosome stalls on a faulty mRNA. Stalled ribosomes activate ER-phagy responses that involve the UFMylation of the ribosomal protein RPL26, which in turn induces the recruitment of a multimeric complex containing the soluble ER-phagy receptor C53, the UFMylation ligase UFL1, and its adaptor DDRGK1, which is an integral ER membrane protein. This event triggers the macro-autophagic delivery of the dysfunctional translocon to lysosomes for degradation (FIGURE 20) (231, 278, 282, 327, 328).

##### 3.3.2.4. pathogen invasion.

Activation of the ER membrane protein STING by cyclic di-AMP released by intracellular Gram-positive bacteria triggers ER stress, which inactivates mTORC1, eliciting autophagy (260). Enhanced ER-phagy responses promote the turnover of ER portions containing the ER stress sensors PERK and IRE1 (sect. 3.3.1.2.1), thus preventing the activation of ER-stress-induced proapoptotic pathways (260, 465). ER-phagy may also be initiated by STING ubiquitylation and recognition by p62 (466) or by direct binding of STING to Atg8/LC3 proteins (FIGURE 20) (259, 262). 

In plants, ER-centric activation of ER-phagy responses has been observed in *A. thaliana* cells infected with the Turnip yellows virus. The viral protein P0 activates the ER-phagy receptors ATI1 and ATI2 and results in an ATG8-mediated ER-to-lysosome-associated degradation of ER portions containing the antiviral protein Argonaute 1 (AGO1) (FIGURE 20) (298).

##### 3.3.2.5. neurite growth.

The components of the ATG machinery have also been implicated in various forms of secretion. For example, autophagosomes can sequester organelles like mitochondria or specific cytokines and expel them in the extracellular milieu upon fusion with the plasma membrane (467, 468). This noncanonical type of autophagy has been called secretory autophagy. Additionally, lipidated Atg8/LC3 on late endosomes can target specific proteins into their internal vesicles, and upon fusion of the resulting multivesicular bodies with the plasma membranes they are released as extracellular vesicles (467, 468). Very interestingly, it has recently been reported that specific components of the ER-phagy machinery are involved in a pathway that does not result in lysosomal clearance of the ER but in its excretion from cells (469, 470). This happens during neurite growth, which is characterized by a controlled expansion of the ER to produce the lipids and proteins that are required for the enormous expansion of plasma membrane surface, up to 200,000 times, of the neuron cell body. ER expansion is counterbalanced by enhanced ER turnover via ER-phagy, which is sustained by the generation of ER fragments containing RTN proteins such as RTN1, RTN3S, and RTN4, and the ER-phagy receptors ATL3 and CALCOCO1. These Atg8/LC3-positive ER fragments appear to be engulfed by late endosomes through a process reminiscent of type III micro-autophagy, and they are eventually excreted from cells upon VAMP7-dependent fusion of the resulting multivesicular bodies with the plasma membrane (469, 470).

## 4. LYSOSOMAL CLEARANCE OF THE ER AND RELATED HUMAN DISEASES

Several ER-phagy receptors have multiple established functions in cells, and only recently has their role in ER turnover been reported (sect. 3.2). Mutations in their genes have been linked to a number of diseases, and emerging data have shown that some of them are probably the target of viral and bacterial pathogens (TABLE 2). In most of the cases, the exact contribution of dysfunctional ER-phagy to the pathophysiology of the disease remains to be established, although altogether the reports point to an important function of ER-phagy in human biology, especially in the maintenance of the homeostasis of the nervous systems (47, 48).

**Table 2.** Genes specifically involved in ER-phagy that have been associated with diseases

Type of Disease	ER-Phagy Receptor or Associated Proteins	Specific Pathology
Neurological disorders	FAM134B	HSAN2B
	ATL3	HSAN1
	ATL1	HSP, HSN I
	RTN3L	Alzheimer’s disease
	CALCOCO1	Tardive dyskinesia, panic disorder
	SEC24C	
	LNPK	
	TEX264	(Defect in DNA repair)
Cancer	FAM134B	Colorectal, esophageal squamous cell, hepatocellular, and pancreatic cancers
	CALCOCO1	Colorectal cancer
	SEC62	Prostate, cervical, thyroid, hepatocellular, and lung cancers
	TEX2643	Renal cell and colorectal cancers
Infections	FAM134B	DENV, ZIKV
	SEC62	FMDV
	ATL1	DENV, ZIKV, WNV
	ATL2	DENV, ZIKV, WNV
	ATL3	DENV, ZIKV, WNV
	RTN3	DENV, ZIKV, WNV, HCV
Others	FAM134B	Allergic rhinitis
	FAM134B	Vascular dementia

See glossary for abbreviations.

### 4.1. ER-Phagy Receptors and Neurological Disorders

A few ER-phagy receptors and adaptors have been linked to neurodegenerative and/or neurodevelopment disorders. Mutations in *FAM134B* result in hereditary sensory and autonomic neuropathy 2B (HSAN2B), an autosomal recessive disorder characterized by the loss of myelinated and unmyelinated fibers, which leads to an impairment of pain, temperature, and touch sensation (hypo- and areflexia) upon degeneration of dorsal root and autonomic ganglion cells (204, 471, 472). Although the pathophysiology of HSAN2B has not been directly correlated with a defect in ER-phagy, the truncation at Glu145 found in a subset of patients (204) removes part of the reticulon-homology domain and the LIR motif, thereby substantially impairing the function of FAM134B in ER fragmentation and subsequent delivery to lysosomes (200). Moreover, ER tubules are enlarged in the dorsal root ganglia tissue of *fam134b*−/− mice and FAM134B-depleted U2OS cells (200), which may be indicative of dysfunctional ER-phagy. Intriguingly, SEC24C has been implicated in the maintenance of neuronal homeostasis (473), and mutations in *LNPK* cause complex neurodevelopmental syndrome (474).

Mutations in *ATL3*, in contrast, lead to hereditary sensory and autonomic neuropathy 1 (HSAN1), another disorder primarily affecting the peripheral nervous system (101, 475, 476). Importantly, HSAN1-associated mutations Y192C, which localizes in the GIM of ATL3, and P338R disrupt the binding between ATL3 and GABARAP and impair ATL3 function in ER-phagy (214) but also directly or indirectly other ER functions (477). *ATL1* is one of the genes that is mostly found to be mutated in the early-onset forms of autosomal dominant hereditary spastic paraplegia (HSP) (478, 479). Interestingly, mutations in this gene have also been associated with hereditary sensory neuropathy type I (HSN I) (480). Although the role of ATL1 in ER-phagy still needs investigation, this protein binds GABARAP (214) and a HSP-associated mutation, Y196C, which localizes in ATL1’s GIM, disrupts ATL1-GABARAP association (481).

RTN3L, which is abundant in neurons, has been linked to Alzheimer’s disease, which is characterized by the accumulation of amyloid beta-protein (Abeta) deposits in the brain. Inhibiting the activity of the beta-amyloid converting enzyme 1 (BACE1) or reducing levels of BACE1 in vivo decreases the production of Abeta. The four members of the RTN protein family are binding partners of BACE1 (482, 483). In the brain, BACE1 mainly colocalizes with RTN3 in neurons, and an increase in the expression of any RTN protein substantially reduces the production of Abeta (482, 483). Conversely, lowering the expression of RTN3 increases the secretion of Abeta, suggesting that this RTN protein is a negative modulator of BACE1 (482–484). Indeed, RTN3 interacts with BACE1 and inhibits its activity, which results in decreased production of Abeta (482, 484). RTN3 overexpression also reduces axonal transport of BACE1, decreasing the level of this secretase in axonal terminals and leading to a reduction in Abeta deposition (485, 486). The relevance of RTN3 in Alzheimer’s disease is also underlined by a study that found multiple RTN3 variants in patients with sporadic early and late onset of this disease (487). To date, however, there is no evidence suggesting a link between RTN3L function in ER-phagy and its role in modulating BACE1 activity, especially because the cytosolic tail of RTN3 containing the LIR motifs appears not to be involved in BACE1 activity modulation (488).

Tardive dyskinesia is a persistent and potentially debilitating condition characterized by repetitive involuntary movement of orofacial regions and extremities, which is largely caused by antipsychotic treatment. Although commonly observed in patients with schizophrenia, tardive dyskinesia can occur in individuals with other psychiatric disorders. Single-nucleotide polymorphisms in *CALCOCO1* appear to confer threefold increase in tardive dyskinesia risk (489). Single-nucleotide polymorphisms in *CALCOCO1* have also been associated with panic disorder, an anxiety disorder characterized by panic attacks and anticipatory anxiety (490).

### 4.2. ER-Phagy Receptors and Tumors

FAM134B acts as a tumor suppressor and inhibits cancer growth and proliferation both in vitro and in vivo (491–494). In addition, *FAM134B* mutations or expression alterations are common in patients with colorectal cancer (CRC), and they are associated with the biological aggressiveness of these cancers (491, 493, 495, 496). In colon cancer cells, it has been found that FAM134B interacts with EB1/MAPRE2 (497), a key factor in the organization of the microtubule cytoskeleton, by binding to the plus-ends of microtubules and serving as a platform for several interacting proteins that control microtubule dynamics. Interestingly, suppression of FAM134B leads to a significant upregulation of EB1, which appears to promote WNT/β-catenin signaling pathways that probably result in colorectal carcinogenesis (497). FAM134B mutations and/or expression have been linked with the development of other tumors as well, including esophageal squamous cell carcinoma (220, 498), hepatocellular carcinomas (499), and pancreatic cancer (500). TEX264 is also a marker protein for CRC (501), and, like FAM134B, its transcripts are strongly induced in glioblastoma cells exposed to pharmacological treatments inducing ER stress and autophagy-induced cell death (217–219).

CALCOCO1 expression has also been found to be altered in CRC and breast cancer (502–504), and this may also be linked to an alteration in the WNT/β-catenin signaling pathways but through an ill-described function of CALCOCO1 in transcription (502, 505–508).

Mutations, amplification, and overexpression of *SEC62*, but also the other subunits of the translocon, i.e., *SEC61* and *SEC63*, have been linked to tumorigenesis (356, 509). In particular, SEC62 levels are frequently altered in prostate, cervical, thyroid, hepatocellular, lung, and other cancers (356, 509, 510). Increased levels of SEC62 confer to these tumors higher metastatic and invasive potential, higher ER stress tolerance, and lower sensitivity to ER stress-induced cell death, with the ultimate negative consequence of reduced patient survival (510). Since an elevated *SEC61* and *SEC63* expression does not accompany that of *SEC62* in tumors (509, 511, 512), it appears that a change in protein translocation activity into the ER is not the etiological factor triggering tumorigenesis. This observation may suggest that the role of SEC62 in ER-phagy is what influences tumorigenesis. However, SEC62 levels also regulate calcium efflux from ER, and consequently other non-mutually exclusive scenarios are possible as well (509, 513).

### 4.3. ER-Phagy Receptors and DNA Repair

Topoisomerase 1 (TOP1) regulates topology of DNA to ensure efficient DNA replication and transcription. The stabilization of a catalytic intermediate between TOP1 and DNA leads to the formation of highly cytotoxic complexes, known as TOP1 cleavage complexes, that can cause genome instability and, if not eliminated, neurological disorders. A study aiming at understanding how cells eliminate TOP1 cleavage complexes found that TEX264 is a central player in this process (514, 515). TEX264 recognizes both unmodified and SUMO1-modifed TOP1 and initiates TOP1 cleavage complex repair by recruiting the valosin containing protein (VCP)/p97 ATPase and the SprT-like NH_2_-terminal domain (SPRTN) metalloprotease. The colocalization between these repair complexes and DNA replication forks indicates that a subpopulation of TEX264 is present on the inner surface of the nuclear membrane (514, 515).

Thus, TEX264 acts as an ER-phagy receptor at both the ER and nuclear membrane facing the cytosol (for ER-phagy) or the inner nuclear membrane facing the nucleus (for DNA repair). Whether there is an overlap between these two distinct roles remains unknown (515). Several lines of evidence indicate that autophagy contributes to the maintenance of genome stability through the degradation of nuclear proteins, micronuclei, and cytosolic chromatin fragments (164, 174, 516–518). Moreover, autophagy also mediates the selective turnover of nuclear components in yeast and mammalian cells through a process that often is referred to as nucleophagy and considered a specific type of ER-phagy (174, 518, 519). Thus, a speculative idea is that the nuclear presence of ER-phagy receptors such as TEX264 but also CCPG1 (163) may help to coordinate the selective degradation of intranuclear components during nucleophagy.

### 4.4. ER-Phagy Receptors and ER Storage Disorders

Misfolded proteins generated inside the ER are typically translocated across the ER membrane and are degraded by cytosolic proteasomes (sect. 3.3.2.2.1). Some misfolded proteins fail to be turned over by this system. Recent evidence has revealed that ER-to-lysosome-associated degradation programs can eliminate the ER subdomains containing proteins that fail to enter ERAD pathways (sect. 3.3.2.2.2), which can accumulate because of genetic mutations and lead to a series of pathologies collectively known as ER storage diseases (449). Overproduction and/or specific stresses/signals can also overload the ER with native but unfolded proteins, including collagen and immunoglobulins (246). In these situations as well, ER-phagy appears to be important to maintain ER homeostasis (449). Thus, although there is currently no direct link, one prediction is that genetic mutations in ER-phagy receptors or associated proteins could lead to an ER storage disease.

### 4.5. ER-Phagy Receptors and Pathogens

As numerous pathogens are not sophisticated microorganisms that can propagate autonomously, they rely on the subversion of either entire pathways or part of their components in host cells to multiplicate and spread. Host cells, however, often activate defensive mechanisms aimed at clearing invading pathogens. Thus, a conspicuous number of microbes have devised strategies to inhibit or at least weaken immune responses or other protective mechanisms to guarantee their intracellular survival. As ER-phagy has the potential of detoxifying the ER from microbial components, it would not be surprising that several pathogens have developed ways to subvert this process.

The first report showing that this is indeed the case is that on the interaction between flaviviruses and FAM134B (520). Infection of cells with Dengue virus (DENV), Zika virus (ZIKV), and West Nile virus (WNV) leads to the cleavage of FAM134B but not that of other ER-resident proteins, including ATL2 and ATL3. The NS3 protease and its NS2B cofactor expressed by these flaviviruses are required for the specific processing of FAM134B, and the resulting fragment is unable to sustain ER-phagy (520). Interestingly, NS3 colocalizes with FAM134B, and this is more accentuated in cells expressing a variant of FAM134B that cannot be cleaved by the flaviviral proteases. Whether colocalization between FAM134B and NS3, and possibly other flaviviral proteins, leads to their lysosomal degradation and whether this occurs in infected cells remain to be determined. Nonetheless, depletion of FAM134B promotes the infection of Dengue and Zika virus, indicating that the FAM134B processing has a beneficial impact for flavivirus multiplication in host cells. In another study, the same laboratory showed that BPI fold-containing family B member 3 (BPIFB3), an autophagy regulator that negatively controls enterovirus replication (521), functions as a positive regulator of DENV and ZIKV infection and that its depletion inhibits the formation of viral replication organelles (522). BPIFB3 knockdown enhances FAM134B-dependent ER-phagy, leading to an increase of ER turnover and the suppression of viral replication. The antiviral effects of BPIFB3 depletion can be reversed with the silencing of FAM134B, but not that of SEC62 or RTN3, suggesting a specific role for BPIFB3 in regulating FAM134B-mediated ER-phagy (522). Intriguingly, there are also data suggesting that FAM134B-dependent ER-phagy may also be important in limiting Ebola virus replication (523).

Although it appears that several Gram-negative bacteria trigger a FAM134B- and SEC62-independent ER-phagy process that is induced by the STING-dependent cell-autonomous response (260), the interaction between flavivirus and FAM134B remains the only documented case to date in which direct subversion of the ER-phagy machinery by a pathogen has been revealed with molecular detail. Nonetheless, there is evidence scattered throughout the literature that suggests that this could be not an isolated case and other viruses may hijack ER-phagy receptors. For example, the infection of foot-and-mouth disease virus (FMDV), a positive-strand RNA virus from the picornavirus family, triggers the expression of SEC62, which acts as an important antiviral factor by upregulating the IRE1-retinoic acid-inducible gene 1 (RIG-I)-dependent antiviral innate immune responses through an unknown mechanism, and FMDV evades this antiviral host defense mechanism by downregulating IRE1 levels (524–526). These observations may indicate that ER-phagy could be part of an immune response helping cells to cope with infections. However, viruses, especially positive-strand RNA viruses, remodel host cell membranes to establish their replication centers and/or assemble their viral particles. As a result, it cannot be excluded that SEC62 could have a role in this context independently of its function in ER-phagy. This could also explain the relevance of ATL1, ATL2, ATL3, and RTN3 in the replication and/or virion assembly of flaviviruses such as DENV, ZIKV, and WNV (527–529), as well as that of RTN3 in enterovirus 71 propagation (530). That is, the ER membrane-modifying properties of these proteins are essential for the infection cycle of these viruses. Interestingly, RTN3 has been found to impair the replication of hepatitis C virus (HCV), another flavivirus, through binding to virally encoded NS4B (94). Consistently, RTN3 depletion promotes HCV propagation. Thus, a possibility is that RTN3 restricts HCV infection by enhancing the degradation of one or more viral factors. This speculation, however, needs further validation also, because another laboratory has found that RTN3 knockdown impairs HCV infection (531).

Finally, it has been shown that mouse hepatitis virus (MHV), the prototype virus for the study of coronaviruses (CoV), coopts the ERAD tuning pathway operating in mammalian cells for lysosomal clearance of select ERAD factors (352–355), to generate the ER-derived membranous platforms involved in viral RNA replication and transcription (532). In agreement with this notion, these platforms are positive for LC3 proteins and for the ERAD factors EDEM1 and OS-9. Importantly, downregulation of LC3 proteins, but not inactivation of the autophagy machinery, protects cells from CoV infection. This highlights the relevance of the ERAD tuning pathway for the coronaviral life cycle (532).

### 4.6. Other Diseases

Allergic rhinitis is a common airway disease in which allergen exposure triggers an IgE-mediated immune response. The typical symptoms include nasal itchiness, rhinorrhea, sneezing, and progressive blockage of the inflamed nasal passages. The disease is driven by a complex interplay of various leukocytes. T regulatory cells are central to the prevention or attenuation of proinflammatory immune responses, and the cell surface expression of the ectonucleosidase CD39/ENTPD1, which hydrolyzes extracellular ATP, is key for the exertion of their function. It has been shown that a genetic polymorphism located in the promoter region of CD39 leads to a variation in its cell surface expression in T regulatory cells, but on its own this polymorphism had no direct impact on risk of allergic rhinitis (533). The disease risk, however, has been linked through an epistatic interaction FAM134B (533).

Vascular dementia is a general term describing problems with reasoning, planning, judgment, memory, and other thought processes caused by brain damage from impaired blood flow to the brain. In this context as well, FAM134B appears to have a strong synergistic epistasis with tumor necrosis factor receptor superfamily, member 19 (TNFRSF19) in the susceptibility to vascular dementia (534).

## 5. CONCLUDING REMARKS

Although the first report about ER-phagy receptors in plants was only published 10 years ago (294) and the identification of ER-phagy receptors in mammalian cells and yeast is even more recent (163, 164, 186, 200, 214, 228, 236, 248–250, 278, 293), massive progress has been made in identifying additional components of the ER-phagy pathways and situations in which ER-phagy is induced. However, how the different physiological and pathological cues engage specific ER-phagy machineries as well as the precise mechanisms of ER fragmentation and subsequent sequestration into autophagosomes, lysosomes, and/or endosomes are central questions that are awaiting a response.

Although somehow delayed, the mechanistic dissection of ER-phagy is following an evolution like the one that mitophagy went through. Mitophagy was thought to be exclusively involved in the degradation of damaged or excess mitochondria and exploited by cells under conditions of starvation to contribute to the generation of an internal pool of nutrients via bulk autophagy. More recently, it has become evident that mitophagy can modulate the transition from a respiratory to a nonrespiratory metabolism and, by doing so, it also participates in cell stemness maintenance, cell differentiation and development, and immunity (535). On the same line, ER-phagy, which contributes nutrients to starving cells as well (sect. 3.3.1), has also been shown to be selectively induced to eliminate dysfunctional and superfluous ER (sect. 3.3.2). The role of ER-phagy in cell development and differentiation has been shown in plants, worms, and vertebrates (222, 294, 295, 469, 536), and further studies will certainly uncover new physiological pathways relying on ER-phagy responses.

Morphologically, the ER has a clear compartmentalization in rough and smooth domains, in tubules and sheets, is associated with other organelles, and defines the nuclear envelope (1, 537). Within those areas, however, only defined portions are selected for fragmentation and lysosomal delivery. These portions are probably generated by segregating defective gene products, excess membranes, and other unwanted macromolecules that need to be removed from cells. How these ER subdomains are confined and detached from the bulk ER is poorly understood. ER-phagy receptors are very likely playing a crucial role in these processes, in which they cluster together and engage cytosolic components of the ATG machineries. Clustering and fragmentation could be achieved through posttranslational modifications that lead to the generation or enhanced exposure of a LIR or a LIR-like motif and could result in direct (for ER-phagy receptors containing reticulon-homology domains) or indirect (via interacting with reticulon-homology domains-containing proteins or atlastins) perturbation of the ER membrane (sect. 3.2.4).

A not mutually exclusive scenario is that the posttranslational modifications trigger a liquid or gel phase separation of the soluble and/or membrane-associated content present in the ER, which must be degraded. This clustering would have the double advantage of effectively segregating the material targeted for destruction from the material that must be preserved and relying on a small number of ER-phagy receptors for the turnover of a large number of proteins. This concept is not unprecedented, since p62-positive aggregates targeted by aggrephagy are formed through liquid phase separation (538–540). Moreover, it has been observed that phosphorylation is required for the selective degradation of mitochondrial matrix proteins (541). Liquid phase separation and condensate formation is often regulated through phosphorylation/dephosphorylation (542). Phosphorylation/dephosphorylation-mediating phase separation often takes place within intrinsically disordered domains (542), and, intriguingly, several ER-phagy receptors possess intrinsically disordered domains (sect. 3.2). Thus, liquid phase separation could also be a mechanism underlying ER-phagy receptor clustering and a concomitant packing of bound cargoes into discrete areas of the ER. Interestingly, this hypothesis is indirectly supported by the observation that cargoes undergoing ER-phagy in the ER tubules are in a liquid phase (457). Moreover, a selective type of macro-autophagy for protein condensates formed by endocytic proteins in yeast has been reported (543). In this process, the endocytic protein Ede1 mediates phase separation into condensates at the plasma membrane and functions as a selective autophagy receptor by binding Atg8. These two properties are necessary for Ede1-dependent macro-autophagy (543). Such a mechanism is also evoked by the fact that p62, which is capable of liquid-liquid phase separation (538–540), is an ER-phagy receptor as well (sect. 3.2.1.3).

Why are there so many ER-phagy receptors? The number of ER-phagy receptors is rapidly increasing. To date, ER-phagy receptors are more than a dozen in mammals, more than half a dozen in plants, and three in yeast. A tissue-specific distribution is observed in mammals and plants (sect. 3.2). Within cells, specific receptors are enriched in ER subdomains, with some located in the three-way junctions, some in tubuli, and others in the sheets (sect. 3.2). Thus, one possibility is that ER-phagy receptor heterogeneity allows appropriate survey of the subcompartments of the ER. An additional advantage of having several ER-phagy receptors is to have a quality control system with sufficient flexibility to detect and deal with sudden unpredicted dysfunctions and/or damages caused by a peculiar stress, alterations/mutations, or the arrival of components from an invading pathogen.

Fundamentally, all processes ensuring lysosomal ER turnover begin with a physical separation of the portion of the ER to be cleared from the bulk ER but then diverge when it comes to the delivery mechanism of these ER portions to the compartments of the endolysosomal system. The diversity of the events that regulate ER turnover after the fragmentation of the ER portions is a fascinating topic for future research. A related question is why there are three different ER-phagy mechanisms (FIGURE 7). A possible scenario would be that having three different ER-phagy mechanisms would equip cells with an alternative solution to eliminate cytotoxic components from the ER and survive in those situations in which one or two of the degradative pathways are compromised.

Although mutations in genes encoding for the ER-phagy receptors and ER-phagy-associated proteins have been linked to several pathologies, a causative connection to dysfunctional ER-phagy remains to be established in most of the cases. The severity and the type of disease vary depending on the mutated gene (sect. 4). This is probably not exclusively due to the type of genetic mutation, e.g., point or missense mutation, but also the tissue distribution of the ER-phagy receptors that make them more relevant in specific organs. For example, whereas SEC62, TEX264, and ATL3 are ubiquitous, CCPG1 is principally present in the pancreas, stomach, kidney, and liver (163). FAM134B is also ubiquitous but expressed in high levels in the brain and peripheral neurons (200). In contrast, the FAM134B-2 isoform is more abundant in the kidney, liver, spleen, and white adipose tissue (208, 221). Consistent with this notion, the absence of each ER-phagy receptor has a different consequence in a multicellular organism. For example, deletion of *FAM134B* in mice affects neurons and the skeleton (200, 222), whereas that of *CCPG1* and *FAM134B-2* alters the functions of the exocrine pancreas and liver, respectively (163, 208). Of note, the severity of the phenotype and the tissue affected may be influenced by the total or partial functional redundancy with one or more other ER-phagy receptors, which can also be different from tissue to tissue.

In conclusion, major pioneering discoveries have been made in the field of ER-phagy during the past decade by unveiling several ER-phagy pathways, players, and physiological roles. This is probably just the tip of the iceberg, and it is predictable that the next years will shed light on multiple molecular principles of the ER-phagy pathways. In addition to generating new important fundamental knowledge, these advances will provide new insight into the pathophysiology of a few devastating diseases caused by mutations in ER-phagy-associated genes and possibly also clues on possible therapeutic avenues to treat them. Autophagy induction has been shown to have the potential to cure, or at least delay, the onset of specific diseases by degrading cytotoxic components (52, 55, 56). In this context, the modulation of selective types of autophagy has the added value of a more precise intervention, which would avoid the conceivable side effects of a bulk approach (544–546). Thus, the fundamental knowledge about how to enhance ER-phagy either physiologically or pharmacologically, by identifying drug targets, could provide strategies to treat diseases affecting the ER, like for example the ER storage disorders.

## 6. GLOSSARY

Atg39: Autophagy-related protein 39; integral membrane receptor for ER-phagy and nucleophagy in yeast
Atg40: Autophagy-related protein 40; integral membrane receptor for ER-phagy in yeast
Atg8: Autophagy-related protein 8; yeast protein; see LC3 and GABARAP
ATL: Atlastin
ATL3: Atlastin-3; mammalian integral membrane receptor for ER-phagy
AtSec62: Plant integral membrane receptor for ER-phagy
ATZ: α_1_-Antitrypsin, Z-variant
Autolysosome: Product of the fusion between autophagosome and lysosome, in which active degradation takes place
Autophagosome: Double-membrane vesicle sequestering cytoplasmic material; its formation depends on ATG proteins
Autophagy: Process leading to the lysosomal degradation of cytoplasmic material, including organelles and invading pathogens
CALCOCO1: Calcium-binding and coiled-coil domain-containing protein 1; mammalian peripheral membrane receptor for ER-phagy
CCPG1: Cell Cycle ProGression 1; mammalian integral membrane receptor for ER-phagy
CMA: Chaperone-mediated autophagy
Cytoplasm: The content of the cytosol, including cell organelles
ER: Endoplasmic reticulum
ER-associated degradation (ERAD): Proteasomal degradation of misfolded proteins from the ER
ER-phagy responses: Cellular responses to physiological or pathological perturbations of ER homeostasis; conserved in all eukaryotic cells, they result in selective or nonselective ER fragmentation and clearance within lysosomes/vacuoles
ER-phagy sites: Specialized ER exit sites involved in the macro-autophagic turnover of ER proteins
ER-phagy/reticulophagy: Selective autophagy of the ER
ER-to-lysosome-associated degradation (ERLAD): Lysosomal (or vacuolar) clearance of ER portions containing misfolded proteins
ESCRT: Endosomal Sorting Complex Required for Transport
FAM134/RETREG: Family with sequence similarity 134/reticulophagy regulators; protein subfamily comprising FAM134B/RETREG1, FAM134A/ RETREG2, FAM134C/RETREG3; mammalian integral membrane receptors for ER-phagy
GABARAP proteins: GABA type A Receptor-Associated Protein; protein subfamily comprising GABARAP, GABARAPL1, and GABARAPL2; see also Atg8
GIM: GABARAP Interacting Motif
Isolation membrane: See Phagophore
LC3 proteins: (Microtubule-associated protein 1) Light Chain 3; protein subfamily comprising LC3A, LC3B, and LC3C; see also Atg8
Lipophagy: Selective autophagy of lipid droplets
LIR: LC3-interacting region
Lysophagy: Selective autophagy of the lysosomes
Lysosome: Umbrella term to define polymorphic, multifunctional membrane-bound organelles, in which acid hydrolases and membrane components are stored
Macro-autophagy: Capture of cytoplasmic content by double-membrane autophagosomes
Macro-ER-phagy: Sequestration of ER portions by autophagosomes
MDV: Mitochondrial-derived vesicle
Micro-autophagy: Direct capture of cytoplasmic content by lysosomal compartments
Micro-ER-phagy: Direct sequestration of ER portions by endosomes/lysosomes/vacuoles
Mitophagy: Selective autophagy of mitochondria
Nucleophagy: Selective autophagy of part of the nucleus
Organellophagy: Organelle-selective autophagic processes
Pexophagy: Selective autophagy of the peroxisomes
Phagophore: Membranous cistern that is the precursor structure of the autophagosome
PtdIns3P: Phosphatidylinositol-3-phosphate
Recov-ER-phagy: ER-phagy induced during recovery from acute ER stresses
REEP: Receptor Expression-Enhancing Protein
RTN: Reticulon
RTN3L: Reticulon 3L; mammalian integral membrane receptor for ER-phagy
SEC62: Component of the SEC61 protein translocation channel and integral membrane receptor for ER-phagy
SNAREs: Soluble *N*-ethylmaleimide-Sensitive Factor Attachment Protein Receptor proteins; mediate vesicle fusion
TEX264: Testis expressed gene 264; mammalian integral membrane receptor for ER-phagy
TFE3: Transcription Factor binding to IGHM Enhancer 3; regulates lysosome biogenesis, autophagy, and energy metabolism
TFEB: Transcription Factor EB; regulates lysosome biogenesis, autophagy, and energy metabolism
Ub: Ubiquitin
UPRs: Unfolded protein responses; cellular responses to physiological or pathological perturbations of ER homeostasis; conserved in all eukaryotic cells, they result in expansion of the ER and of its content in ER chaperones, folding enzymes, biosynthetic factors
Vacuole: Yeast and plant counterpart of the mammalian lysosome

## GRANTS

M.M. is supported by the Swiss National Science Foundation (31003A-163063), Eurostars (E! 113321_CHAPERONE), Innosuisse (35449.1 IP-LS), the AlphaONE Foundation (488078), the Foundation for Research on Neurodegenerative Diseases, and the Comel and Gelu Foundations. F.R. is supported by ZonMW VICI (016.130.606), ZonMW TOP (91217002), ALW Open Program (ALWOP.310), and Marie Skłodowska-Curie Cofund (713660) and Marie Skłodowska Curie ETN (765912) grants “into” F.R. are supported by ZonMW TOP (91217002), Open Competition ENW-KLEIN (OCENW.KLEIN.118), SNSF Sinergia (CRSII5_189952), and Novo Nordisk Foundation (0066384) grants.

## DISCLOSURES

No conflicts of interest, financial or otherwise, are declared by the authors.

## AUTHOR CONTRIBUTIONS

F.R. and M.M. prepared figures; F.R. and M.M. drafted manuscript; F.R. and M.M. edited and revised manuscript; F.R. and M.M. approved final version of manuscript.
